# Supplementary material for: Cell-free biosynthesis and engineering of ribosomally synthesized lanthipeptides
Source: Nat Commun. 2024 May 21;15:4336. doi: 10.1038/s41467-024-48726-y (PMC11109155; doi:10.1038/s41467-024-48726-y)
Supplement: Supplementary file 1 — Supplementary Information [file 41467_2024_48726_MOESM1_ESM.pdf]

Supplementary Information

Cell-free biosynthesis and engineering of ribosomally synthesized lanthipeptides

Wan-Qiu Liu[1,#], Xiangyang Ji[1,#], Fang Ba[1], Yufei Zhang[1], Huiling Xu[1], Shuhui Huang[1], Xiao Zheng[1], Yifan Liu[1,2,3*], Shengjie Ling[1,2,3*], Michael C. Jewett[4,*], and Jian Li[1,2,3*]

[1] School of Physical Science and Technology, ShanghaiTech University, Shanghai 201210, China

[2] State Key Laboratory of Advanced Medical Materials and Devices, ShanghaiTech University, Shanghai 201210, China

[3] Shanghai Clinical Research and Trial Center, Shanghai 201210, China

[4] Department of Bioengineering, Stanford University, Stanford, CA, 94305, United States

[#] These authors contributed equally to this work.

* Corresponding authors.

E-mail addresses: lijian@shanghaitech.edu.cn (J. Li), mjewett@stanford.edu (M.C. Jewett), lingshj@shanghaitech.edu.cn (S. Ling), liuyf6@shanghaitech.edu.cn (Y. Liu).

Supplementary Tables

Supplementary Table 1 Gene sequences used in this study (genes are codon-optimized for *E. coli*).

Gene name	Sequence (5'-3')
sboA	atggcgaagcagcaaatgaacctggtggagatcgaagcgatgaacagcctgcaggagctgaccctggaggagctggacaacgtgctgggtgcgggtg<br>gcggtgttatccagaccattagccacgaatgccgtatgaacagctggcaattcctgtttacctgctgcagc
*sboA*_A-1R	atggcgaagcagcaaatgaacctggtggagatcgaagcgatgaacagcctgcaggagctgaccctggaggagctggacaacgtgctgggtcgtggtg<br>gcggtgttatccagaccattagccacgaatgccgtatgaacagctggcaattcctgtttacctgctgcagc
sboM	atggtgaaaatggagaacaccgaaaagaacaaactgttcaacaagtttacccgtaacttcatccagaccaactttagcgaatacgatatgcaaaactgcga<br>gatcgagttcctggaggacaaagttctggatatttatatgaacaccctgatcctgctgattaacgaaaaacgtctgctgggtatcctgaagggcagcaccag<br>cgaggaacgttacgaatatttcaacgacgtgctgtgcggcaagtacatcattgatgagattgcgaaacgttttccgaagctggttagccgtagcctgaccca<br>gctgaaaaactacctgctgctgtatcgtaaggtggagcaactgttcctgaaagacgttccgaagctgatccagaaccgtttcattagcaaccaatgcgagaa<br>ctttaaaatggaagacctgcagatcaccgtgagcggcgatttccacaacggtagcggcgtttgcatcctgttttacaaaggtgaacgtgtgatttataagggc<br>aaaaacaacttcgcgaaccaactgctgatcgagatttttagcaagctgggtgatgagttcgaggaaagcctggaatttctgccgcgttacatcgactgcggc<br>gattactattgggaggaatatatcgaacacaccagcattcgtaacagcaacatcagcgcgagcgagtactataaacgtttcggttacctgctggcggtggc<br>gtatctgctgaacatcagcgacctgcactttgaaaacattctggcgagcggcgacatcccgaaactggtggacgttgagaccattttcaacctgcagccgta<br>cgagattctgagcgagaccatcgcggacaaagatctgctgaagcgtaacgcggaaagcgttctgctgaccggtctgctgccgctggcgggcagcaacg<br>aggtgttcggtggcgataccagcggtatcctgggtggcaaatttgttggcgaagtgcgtgttattcagaaccaaatgcgtgacgatatcagcgtgaagcgtc<br>aggtggttcgtaaaaccaacaagagccacctgccgttctacatcagcgagggcgaggaacaatactttgaagttaaagactatctgcgtgagctgctgttc<br>ggctttaagaaagtgaccagctacgttgagaaccagaagaacaccttcctgagcatcattgagaaatatagcgaaaagatcgacgttcgtattctgtttcgta<br>acaccaaagattacagcatcgtgcgtaccctgctgctgagcccgaagtatagcgaaaacgacaacatcatcttcgagaagttcaagaacaagctggttaac<br>taccaaagcgatgaactgtgcgagagcgaaattaaacagctggagcaaatggacatcccgtatttcagcatgaagagcaacagctgcgacgtgtacgatc<br>tgtatggtaacgcggtttggagcacctactatagcccgatcgaactgattcgtaagaaaatccagaacctgagcgtgcacatcattgaggaacaatacaaac<br>tgatcgagtttagcattagcagcaccctgcgtctgtataaccaggaagcgctgccgcgtattaacgacgttgaggataaaagctacagcgacagcattctgt<br>ataagggtgtgcagaacatcgttgatcgtctgctgggcgcgatttacctgagcaacagcgaccaaacctgcaactggctgaccctgagcatcaacaacgt<br>ggactgcctggaactgagcccgatggattgcaccctgtatggtggcctgagcggtgttgcgctggcgctgatcgagagcatgaccctgctggaggaaga<br>ttatcgtaagaaagtgcgtagcgttctgccgtacctgtataacagcattctgaagaaatacgagtatctgaccaacgacagcttctacatgggtcgtatgggc<br>gttctgtataccctgaagaaactgagcaaagaagtgggcaaggacctggataactgggtgaacatcgagattgataacgttattagccgtctgaacatcggt<br>gacaccgatttcctgaccggcatcagcagcgttatcccggcgattcgtctggagaacctgaacaaagtgacctttgaggaactgagcgacatctacattaa<br>gcaggcgaaagaaaaggataacttcgtgttctggggtaacgagcaaagcaacaacgtgagcctggcgcacggtaacctgggcatcgaactggcgctgg<br>tgcgtctgattaaagttctggacaacagcaacaacaaggagaccatcagcaccctgctgaagaaagcgattaacttcgacaacctgcagcgtcgtgaaca<br>cggctgggtggataaccgtaattttagccacagcgcgaactggtgccatggtgcgaccggcgttctgctgagccgtatcgacctgtacaaccagctgaaa<br>aacgataagaccgcgaccattagcagcaccgacctgcagcaagatgttattcacagcctgcaagatatcaaagaggtgggtctgaacctggcgaacttca<br>gcctgtgccacggtgtgagcggcaacctgctgaccctggttcacacccagaacttctttgacgaaaacaacatcgtgaacaactacaacctgaagaaattc<br>gttcgtgagaacttctacaagatccacctgtatggtctgcaaaacggctggatgtgcaacttcaacaccaagtacgacagctatgcgctgtttaccggtatca<br>gcggcattctgtacgcgaccaccctgtatctgaaaaacgacatcaagagcgatgtgctgctgccgaacattgtgagcctggttccgcgtggtagctggagc<br>cacccgcagttcgaaaaaggtggcagcggtggcagctggagccacccgcaatttgagaagtaa
sboT	atgggcatgaaaatcattctgcagaacaacgaaagcgactgcctgctggcgtgctacgcgatgctgctgaacaacctgggtctggagatcccgctgtacg<br>aaatttataaagaggacatgattccggcggatggtctgaacgtgagctacctgatcaaactgaacaagcgtttcaaagttggcatcaaagcgtatcgtattct<br>gctgaaggacatcgcggtgtgcctggagaagaacattccggttatcctgcactggaacgataaccactttgtggttctgctgaagatccgtaacaagaaata<br>taccatcattgatccggcgattggtaaaatcacctacagcgaggaagaggtgctgaagcactatagcggtatttgcgtgaccgttttcaaaggcgtggaattt<br>aagcgtatcaaatacagcaacctgttcgttgagcagctgcgtcaaaccattcgtctgaagccggcgatgtattttattctggcgctgggtctgacccaggcg<br>ggcgtgttcctgtttagcatcaccattcgtaacattctggacgttaaatacagcttcatcgcgagcctgctgatgctgatgagcgtggttgtggttcaactgatc<br>agcgtgctgattaagaacgaaaccatcagcgagttcaacaacgactttgatgaaaactacgttaacaagctgttcaaaatgctgctgaacaaaccgctgctg<br>tattttcgtaacatgagcaacggtgtgctgggcgagaaaatcaacctgaagagcaccatccgtgatagcattaccctgaaactgctgccgagcatcgttggt<br>ttcattagcctgtttgtggttattctgtacctgctgaccatcagcaccaagctgaccttcattctgctgatcctgctgatcttctacggcatcgtgagcagcgttct<br>gtataagaaacagaacgaactgaaccacgcgtacctgcaatatctgatcgacttcaacagcgtgctgcagaccgacctggagaacattgatttcatcaaaat<br>tgcgcgtaaggaagagcaaatctttaagagctggaaaagcagcaacaacctggttaccgataaatacagcagcatcctgaaggtggaaaacttcagcca<br>gtttgttggtagcacctttaactatattagcctgagcctgatcctgattctgggcatctacttcaaagcgtattttgaagtgagcatcccggacctgattctgtacc<br>aaaccagcgttagcctgatgattagcttctttgaacagatcaagcaaagcatcttcgagattgcgaaactgcaggtgtatgcggagaagcaaagcgacctgt<br>tcaccaacaacgcgagcatcgaaattccgatcgacaacgagcagagcagcgtgatccgttgcgataaagttaacttcagctacaacaaccaaaagctgta<br>tagcggtatcaacctggaaattctgaagggcgagaaaattgcgatcctgggcgaaagcggtagcggcaaaagcaccctgctgctgctgctgctgggtatg<br>ctgcgttacgacggcgagctgacctatggcgtgagcgatttcaccagccgtattggcgtggttctgcagaacatgaccctggttccggacaccatcctgaa<br>gaacctggattgcgcgccgagcgacgaagatctggagcgtattctgcgtgacaccggtggcgaacgtgtgatcgcgaaactgccgaacaagctgtttag<br>caaggttctgaaaagcggcaagaacctgagcggtggccagattcaaaaactgctgatcagccgtagcctgctgaacaagcgtgacatcattttctgggatg<br>aagcgtttagcaacctggatgagaaaagcaagaaaaagatctacaccgacgttctgagcaacccgttctatagcgataagacgatggtgattgttagccac<br>cagctggacatcgtgaactacgttgatagcatcatttttgtggacaaaagcagcggcgatgttattaaggacacccacgataacctgatctaccgtaaccaa<br>aactatcgtaagctgttcggtctgaaagagaaggtgcacaacgacctggttccgcgtggcagccatcatcaccatcatcaccaccaccaccactaa

Supplementary Table 2 Primers used in this study (F, forward primer; R, reverse primer; Bold letters, mutated codon).

**Primer name**	**Oligonucleotide sequence (5'-3')**	**Application**
*sboA*_pJL1_F	catcatcatcaccatatggcgaagcagcaaatgaac	Template construction for cell-free gene expression
*sboA*_pJL1_R	tgttagcagccggtcgacttagctgcagcaggtaaacag	
*sboM*_pET28a_F	ccgcgcggcagccatatggtgaaaatggagaacac	
*sboM*_pET28a_R	gtggtggtggtgctcgagttacttctcaaattgcgg	
*sboT*_pET28a_F	ccgcgcggcagccatatgatgggcatgaaaatcatt	
*sboT*_pET28a_R	gtggtggtggtgctcgagttagtggtggtggtggtg	
*sboA*_pRSFDuet_F	catcaccacagccaggatccgatggcgaagcagcaaatgaac	Plasmid construction for *in vivo* expression
*sboA*_pRSFDuet_R	cattatgcggccgcaagcttttagctgcagcaggtaaacag	
*sboM*_pRSFDuet_F	catcaccacagccaggatccg atggcgaagcagcaaatgaac	
*sboM*_pRSFDuet_R	cattatgcggccgcaagctt ttagctgcagcaggtaaacag	
*sboA*_A-1R_pRSFDuet_F	gtgctgggtcgtggtggcggtgttatccagaccattagtggaggagctggacaacgtgct gggtcgtggtggcggt	
*sboA*_A-1R_pRSFDuet_R	accgccaccacgacccagcacgttgtccagctcctcca	
*sboA*_G-2R_ pRSFDuet_F	acgtgctgggtcgtgcgggtggcggtgttatccagaccat	
*sboA*_G-2R_ pRSFDuet_R	acaccgccacccgcacgacccagcacgttgtccagctcctc	
*sboA*_Q6A_F	ggtgttatc**gct**accattagccacgaatgccgtatgaac	Site-specific mutagenesis
*sboA*_Q6A_R	gctaatggt**agc**gataacaccgccacccgcacccagcac	
*sboA*_Q6C_F	ggtgttatc**tgc**accattagccacgaatgccgtatgaac	
*sboA*_Q6C_R	gctaatggt**gca**gataacaccgccacccgcacccagcac	
*sboA*_Q6D_F	ggtgttatc**gat**accattagccacgaatgccgtatgaac	
*sboA*_Q6D_R	gctaatggt**atc**gataacaccgccacccgcacccagcac	
*sboA*_Q6E_F	ggtgttatc**gaa**accattagccacgaatgccgtatgaac	
*sboA*_Q6E_R	gctaatggt**ttc**gataacaccgccacccgcacccagcac	
*sboA*_Q6F_F	ggtgttatc**ttt**accattagccacgaatgccgtatgaac	
*sboA*_Q6F_R	gctaatggt**aaa**gataacaccgccacccgcacccagcac	
*sboA*_Q6G_F	ggtgttatc**ggc**accattagccacgaatgccgtatgaac	
*sboA*_Q6G_R	gctaatggt**gcc**gataacaccgccacccgcacccagcac	
*sboA*_Q6H_F	ggtgttatc**cat**accattagccacgaatgccgtatgaac	
*sboA*_Q6H_R	gctaatggt**atg**gataacaccgccacccgcacccagcac	
*sboA*_Q6I_F	ggtgttatc**att**accattagccacgaatgccgtatgaac	
*sboA*_Q6I_R	gctaatggt**aat**gataacaccgccacccgcacccagcac	
*sboA*_Q6K_F	ggtgttatc**aaa**accattagccacgaatgccgtatgaac	
*sboA*_Q6K_R	gctaatggt**ttt**gataacaccgccacccgcacccagcac	
*sboA*_Q6L_F	ggtgttatc**ctt**accattagccacgaatgccgtatgaac	
*sboA*_Q6L_R	gctaatggt**aag**gataacaccgccacccgcacccagcac	
*sboA*_Q6M_F	ggtgttatc**atg**accattagccacgaatgccgtatgaac	
*sboA*_Q6M_R	gctaatggt**cat**gataacaccgccacccgcacccagcac	
*sboA*_Q6N_F	ggtgttatc**aat**accattagccacgaatgccgtatgaac	
*sboA*_Q6N_R	gctaatggt**att**gataacaccgccacccgcacccagcac	
*sboA*_Q6P_F	ggtgttatc**cct**accattagccacgaatgccgtatgaac	
*sboA*_Q6P_R	gctaatggt**agg**gataacaccgccacccgcacccagcac	
*sboA*_Q6R_F	ggtgttatc**cgt**accattagccacgaatgccgtatgaac	
*sboA*_Q6R_R	gctaatggt**acg**gataacaccgccacccgcacccagcac	
*sboA*_Q6S_F	ggtgttatc**agt**accattagccacgaatgccgtatgaac	

*sboA*_Q6S_R	gctaatggt**act**gataacaccgccacccgcacccagcac	
*sboA*_Q6T_F	ggtgttatc**acg**accattagccacgaatgccgtatgaac	
*sboA*_Q6T_R	gctaatggt**cgt**gataacaccgccacccgcacccagcac	
*sboA*_Q6V_F	ggtgttatc**gtt**accattagccacgaatgccgtatgaac	
*sboA*_Q6V_R	gctaatggt**aac**gataacaccgccacccgcacccagcac	
*sboA*_Q6W_F	ggtgttatc**tgg**accattagccacgaatgccgtatgaac	
*sboA*_Q6W_R	gctaatggt**cca**gataacaccgccacccgcacccagcac	
*sboA*_Q6Y_F	ggtgttatc**tat**accattagccacgaatgccgtatgaac	
*sboA*_Q6Y_R	gctaatggt**ata**gataacaccgccacccgcacccagcac	
*sboA*_L20_F	catcatcatcaccatatggcgaagcagcaaatgaac	
*sboA*_L20A_R	gttagcagccggtcgacttagctgcagcaggtaaa**agc**gaattgccagct	
*sboA*_L20C_R	gttagcagccggtcgacttagctgcagcaggtaaa**gca**gaattgccagct	
*sboA*_L20D_R	gttagcagccggtcgacttagctgcagcaggtaaa**atc**gaattgccagct	
*sboA*_L20E_R	gttagcagccggtcgacttagctgcagcaggtaaa**ttc**gaattgccagct	
*sboA*_L20F_R	gttagcagccggtcgacttagctgcagcaggtaaa**aaa**gaattgccagct	
*sboA*_L20G_R	gttagcagccggtcgacttagctgcagcaggtaaa**gcc**gaattgccagct	
*sboA*_L20H_R	gttagcagccggtcgacttagctgcagcaggtaaa**atg**gaattgccagct	
*sboA*_L20I_R	gttagcagccggtcgacttagctgcagcaggtaaa**aat**gaattgccagct	
*sboA*_L20K_R	gttagcagccggtcgacttagctgcagcaggtaaa**ttt**gaattgccagct	
*sboA*_L20M_R	gttagcagccggtcgacttagctgcagcaggtaaa**cat**gaattgccagct	
*sboA*_L20N_R	gttagcagccggtcgacttagctgcagcaggtaaa**att**gaattgccagct	
*sboA*_L20P_R	gttagcagccggtcgacttagctgcagcaggtaaa**agg**gaattgccagct	
*sboA*_L20Q_R	gttagcagccggtcgacttagctgcagcaggtaaa**ctg**gaattgccagct	
*sboA*_L20R_R	gttagcagccggtcgacttagctgcagcaggtaaa**acg**gaattgccagct	
*sboA*_L20S_R	gttagcagccggtcgacttagctgcagcaggtaaa**act**gaattgccagct	
*sboA*_L20T_R	gttagcagccggtcgacttagctgcagcaggtaaa**agt**gaattgccagct	
*sboA*_L20V_R	gttagcagccggtcgacttagctgcagcaggtaaa**aac**gaattgccagct	
*sboA*_L20W_R	gttagcagccggtcgacttagctgcagcaggtaaa**cca**gaattgccagct	
*sboA*_L20Y_R	gttagcagccggtcgacttagctgcagcaggtaaa**ata**gaattgccagct	
linearPF	cctacagcgtgagcattg	Construction of hybrid peptides
linearPR	cgcacccagcacgttgtc	
linearTF	gtcgaccggctgctaaca	
linearTR	cggattcagtcgtcactca	
*lctA*_F1	aaaggcggcagtggagttattcatacaatttctcatgaatgtaatatgaatagctggcaatttgtatttacttgctgctcttaa	
*lctA*_R1	ttaagagcagcaagtaaatacaaattgccagctattcatattacattcatgagaaattgtatgaataactccactgccgccttt	
*lctA*_F	gacaacgtgctgggtgcgaaaggtggtagcggtgtg	
*lctA*_R	tgttagcagccggtcgacttagctgcaacaagtgaa	
*rumA*_F1	ggtaacggcgttctgaagaccatctcccacgaatgcaacatgaacacctggcagttcctgttcacctgctgctaa	
*rumA*_R1	ttagcagcaggtgaacaggaactgccaggtgttcatgttgcattcgtgggagatggtcttcagaacgccgttacc	
*rumA*_F	gacaacgtgctgggtgcgggtaacggcgttctgaag	
*rumA*_R	tgttagcagccggtcgacttagcagcaggtgaacag	
*nuk*_F1	aaaaaaaaatctggcgttatcccgaccgtttctcacgattgccacatgaacagcttccagttcgtgttcacctgctgctcctaa	
*nuk*_R1	ttaggagcagcaggtgaacacgaactggaagctgttcatgtggcaatcgtgagaaacggtcgggataacgccagattttttttt	
*nuk*_F	gacaacgtgctgggtgcg aaaaaaaaatctggcgttatc	
*nuk*_R	tgttagcagccggtcgac ttaggagcagcaggtgaa	
*mutII*_F1	aatcgctggtggcagggcgtagttccgactgtatcttacgaatgtcgtatgaactcttggcagcacgtgttcacttgctgctaa	

*mutII*_R1	ttagcagcaagtgaacacgtgctgccaagagttcatacgacattcgtaagatacagtcgg aactacgccctgccaccagcgatt	
*mutII*_F	gacaacgtgctgggtgcg aatcgctggtggcaggg	
*mutII*_R	tgttagcagccggtcgac ttagcagcaagtgaaca	
*salA*_F1	aaacgtggtagcggttggattgctaccatcaccgatgattgtccgaactccgtatttgtttgc tgctaa	
*salA*_R1	ttagcagcaaacaaatacggagttcggacaatcatcggtgatggtagcaatccaaccgct accacgttt	
*salA*_F	gacaacgtgctgggtgcg aaacgtggtagcggttg	
*salA*_R	tgttagcagccggtcgac ttagcagcaaacaaatac	
Lan1_F1	ggtaacggcgttgtgaccaccatctctcacgaatgcaacatgaacagctggcagttcctg ttcacctgttgctaa	
Lan1_R1	ttagcaacaggtgaacaggaactgccagctgttcatgttgcattcgtgagagatggtggtc acaacgccgttacc	
Lan1_F	gacaacgtgctgggtgcgggtaacggcgttgtgacc	
Lan1_R	tgttagcagccggtcgac ttagcaacaggtgaacag	
Lan2_F1	ggcaacggcgttatcaaaaccatctcccacgaatgcgctatgaacacctggcagttcattt tcacctgctgctcctaa	
Lan2_R1	ttaggagcagcaggtgaaaatgaactgccaggtgttcatagcgcattcgtgggagatggt tttgataacgccgttgcc	
Lan2_F	gacaacgtgctgggtgcgggcaacggcgttatcaaa	
Lan2_R	tgttagcagccggtcgacttaggagcagcaggtgaa	
Lan3_F1	ggcgatggtgttatgcataccctgacccatgaatgtcacatgaacacttggcagttcctgct gacctgttgctaa	
Lan3_R1	ttagcaacaggtcagcaggaactgccaagtgttcatgtgacattcatgggtcagggtatg cataacaccatcgcc	
Lan3_F	gacaacgtgctgggtgcgggcgatggtgttatgcat	
Lan3_R	tgttagcagccggtcgacttagcaacaggtcagcag	
Lan4_F1	aacggtgttatcaccacgattagccacgaatgccacctgaacacctgggccttcgcgttc acctgttgctcttaa	
Lan4_R1	ttaagagcaacaggtgaacgcgaaggcccaggtgttcaggtggcattcgtggctaatcg tggtgataacaccgtt	
Lan4_F	gacaacgtgctgggtgcgaacggtgttatcaccacg	
Lan4_R	tgttagcagccggtcgacttaagagcaacaggtgaa	
Lan5_F1	ggtaacggtgttttccacactatttctcacgaatgttactacaactcctgggctttcattttcac ctgctgctaa	
Lan5_R1	ttagcagcaggtgaaaatgaaagcccaggagttgtagtaacattcgtgagaaatagtgtg gaaaacaccgttacc	
Lan5_F	gacaacgtgctgggtgcgggtaacggtgttttccac	
Lan5_R	tgttagcagccggtcgacttagcagcaggtgaaaat	
Lan6_F1	ggtaacggcgctatccgtaccatcagccatgaatgtcacatgaacacctggcagttcctgt ttacctgttgctcttaa	
Lan6_R1	ttaagagcaacaggtaaacaggaactgccaggtgttcatgtgacattcatggctgatggta cggatagcgccgttacc	
Lan6_F	gacaacgtgctgggtgcgggtaacggcgctatccgt	
Lan6_R	tgttagcagccggtcgacttaagagcaacaggtaaa	
Lan7_F1	ggcaacggtcagtttaacactatttctcatgaatgccactggaacacctggcagttcatgtt cacctgctgttcttaa	
Lan7_R1	ttaagaacagcaggtgaacatgaactgccaggtgttccagtggcattcatgagaaatagt gttaaactgaccgttgcc	
Lan7_F	gacaacgtgctgggtgcgggcaacggtcagtttaac	
Lan7_R	tgttagcagccggtcgacttaagaacagcaggtgaa	
Lan8_F1	cgcaaatctggtgtcatcaacaccgtttcccacgaatgcaacatgaactcttggcagttcgt gttcacctgctgcgcgtaa	
Lan8_R1	ttacgcgcagcaggtgaacacgaactgccaagagttcatgttgcattcgtgggaaacggt gttgatgacaccagatttgcg	
Lan8_F	gacaacgtgctgggtgcg cgcaaatctggtgtcatc	

Lan8_R	tgttagcagccggtcgacttacgcgcagcaggtgaa	
Lan9_F1	gataaaggtcagttcgacaccatctctcacgaatgtcattggaacacctggcagtttctgtttacctgttgctcctaa	
Lan9_R1	ttaggagcaacaggtaaacagaaactgccaggtgttccaatgacattcgtgagagatggtgtcgaactgacctttatc	
Lan9_F	gacaacgtgctgggtgcggataaaggtcagttcgac	
Lan9_R	tgttagcagccggtcgacttaggagcaacaggtaaa	
Lan10_F1	ggtaacggtgtaatcaaaactattagccatgaatgccacatgaactcctggcagtggattttcacctgctgctcttaa	
Lan10_R1	ttaagagcagcaggtgaaaatccactgccaggagttcatgtggcattcatggctaatagttttgattacaccgttacc	
Lan10_F	gacaacgtgctgggtgcgggtaacggtgtaatcaaa	
Lan10_R	tgttagcagccggtcgacttaagagcagcaggtgaa	
Lan11_F1	ggttctggcgtaatcaagactatcagccacgaatgccacatgaactcttggcaggcgatcttcacttgctgctcctaa	
Lan11_R1	ttaggagcagcaagtgaagatcgcctgccaagagttcatgtggcattcgtggctgatagtcttgattacgccagaacc	
Lan11_F	gacaacgtgctgggtgcgggttctggcgtaatcaag	
Lan11_R	tgttagcagccggtcgacttaggagcagcaagtgaa	
Lan12_F1	ggtaacggtgttgtttataccctgactcatgaatgtaacctggcgacctggacgaaaaaactgaaatgctgctaa	
Lan12_R1	ttagcagcatttcagtttttcgtccaggtcgccaggttacattcatgagtcagggtataaacaacaccgttacc	
Lan12_F	gacaacgtgctgggtgcgggtaacggtgttgtttat	
Lan12_R	tgttagcagccggtcgacttagcagcatttcagttt	
*degP*_H1F	ttgcagcattacacgtcttggggtcattagcggattattag	Construction of homologous fragments for gene deletion
*degP*_H1R	tccagcctacacaatcgctcctcgattaacagataacgca	
*degP*_H2F	gatattcatatggaccatgggggttctccttacaatctgtg	
*degP*_H2R	gacatgggaattagccatgcctgcgccaacaagtgcatca	
*ydgD*_H1F	ttgcagcattacacgtcttggcaggcggctaaaaagcatcat	
*ydgD*_H1R	tccagcctacacaatcgctccgcatatcacactctggtgagta	
*ydgD*_H2F	gatattcatatggaccatggccggttttcgcgacaagctggatc	
*ydgD*_H2R	gacatgggaattagccatgcgcgtaaacctgaactggaggtga	
*ypdE*_H1F	ttgcagcattacacgtcttgcgcttgccagcacattgccgtacagt	
*ypdE*_H1R	tccagcctacacaatcgctcgcccttattcaacgtcttacgcgtga	
*ypdE*_H2F	gatattcatatggaccatgggatctgccgcacttcctgttccgag	
*ypdE*_H2R	gacatgggaattagccatgcctgcaggcacagctcgcagcaatc	
*degQ*_H1F	ttgcagcattacacgtcttgcggcatcattattggtgctgtg	
*degQ*_H1R	tccagcctacacaatcgctccggctaacaatgagacaccagatta	
*degQ*_H2F	gatattcatatggaccatggccgggcatcaggcttacgtgtgat	
*degQ*_H2R	gacatgggaattagccatgcccaccagcaatgcttcaaatacacga	
*hslV*_H1F	ttgcagcattacacgtcttggtgggatcagcacgtcgagaatacgt	
*hslV*_H1R	tccagcctacacaatcgctcggcgacatttgcatctataccaacca	
*hslV*_H2F	gatattcatatggaccatggggcgtacgcttactatagttgtcac	
*hslV*_H2R	gacatgggaattagccatgcggcttcttctcagcagccgtacca	
*pepN*_H1F	ttgcagcattacacgtcttggggcagaaagccactgatat	
*pepN*_H1R	tccagcctacacaatcgctccagtctattcctgttgcgta	
*pepN*_H2F	gatattcatatggaccatgggggctggaaaatctctctggcgat	
*pepN*_H2R	gacatgggaattagccatgcgaccgggattattgttgcttga	
pkD4_attP/attB_F	agaaggccatcctgacggat	
pkD4_attP/attB_R	gcccttagagcctctcaaag	
*degP*_F	gggtcattagcggattattag	Deletion cassette amplification
*degP*_R	cctgcgccaacaagtgcatca	
*ydgD*_F	gcaggcggctaaaaagcatcat	

*ydgD*_R	cgcgtaaacctgaactggaggtga	
*ypdE*_F	cgcttgccagcacattgccgtacagt	
*ypdE*_R	cctgcaggcacagctcgcagcaatc	
*pepN*_F	gggcagaaagccactgatat	
*pepN*_R	cgaccgggattattgttgcttga	
*degQ*_F	cggcatcattattggtgctgtg	
*degQ*_R	cccaccagcaatgcttcaaatacacga	
*hslV*_F	gtgggatcagcacgtcgagaatacgt	
*hslV*_R	cggcttcttctcagcagccgtacca	
*kan*_MR	cctcgtcctgcagttcattca	Deletion verification
*degP*_veriF	gccatccagatgtcgagcagct	
*pepN*_veriF	ctcgcgtaaccagttaagatagt	
*ydgD*_veriF	cagcataaagcagcacctg	
*ypdE*_veriF	aggctgtcgctttgtaacagggtca	
*degQ*_veriF	gggttagtcgtcggcatcat	
*hslV*_veriF	gacggttcctgctgctgttcggt	

Supplementary Table 3 Plasmids constructed in this study.

Plasmid	Antibiotic	Application
pJL1_*sboA*	50 µg/mL kanamycin	Templates used for cell-free gene expression
pET28a-*sboM*		
pET28a-*sboT*		
pJL1-*sboA*_Q6/L20 (a total of 53 mutated plasmids)		
pRSFDuet-1-*sboA*-*sboM*		Plasmids used for *in vivo* expression
pRSFDuet-1-*sboA*_A-1R-*sboM*		
pRSFDuet-1-*sboA*_G-2R-*sboM*		
pRSFDuet-1-*sboA*_A-1R_Q6H-*sboM*		
pRSFDuet-1-*sboA*_A-1R_L20I-*sboM*		
pRSFDuet-1-*sboA*_A-1R_L20M-*sboM*		
pRSFDuet-1-*sboA*_A-1R_L20I_Q6H-*sboM*		
pRSFDuet-1-*sboA*_A-1R_L20M_Q6H-*sboM*		
pRSFDuet-1-*sboA*_A-1R_L20M_Q6K-*sboM*		
pKD4_*attP*/*attB*-*degP*	50 µg/mL kanamycin 100 µg/mL ampicillin	Plasmids used for protease/peptidase deletion
pKD4_*attP*/*attB*-*degQ*		
pKD4_*attP*/*attB*-*hslV*		
pKD4_*attP*/*attB*-*pepN*		
pKD4_*attP*/*attB*-*ydgD*		
pKD4_*attP*/*attB*-*ypdE*		
pKD46	100 µg/mL ampicillin	Expression of λ-Red recombinases
pYF2	100 µg/mL ampicillin	Expression of TP901-1 integrase

Supplementary Table 4 Proteases and peptidases deleted in the genome of *E. coli* BL21 Star (DE3).

Gene	Protein product	Activity	Reference
degP	Serine endoprotease, DegP	DegP and DegQ may degrade transiently denatured and unfolded proteins, which accumulate in the periplasm (Cleavage sites: Val/Xaa or Ile/Xaa).	1, 2
degQ	Serine endoprotease, DegQ		1, 2
ydgD	Serine endoprotease, YdgD	-	UniProt: P76176
hslV	ATP-dependent protease subunit, HslV	Protease subunit of a proteasome-like degradation complex believed to be a general protein degrading machinery.	3
pepN	Aminopeptidase N, PepN	PepN is responsible for the majority of the aminopeptidase activity in *E. coli* and that it prefers to cleave basic and small amino acids at the amino terminus of substrates.	4
ypdE	Aminopeptidase, YpdE	YpdE has a broad aminopeptidase activity on non-blocked peptides by progressively cleaving amino acids off the peptide substrate. Aminopeptidase activity stops at the residue before the first proline in the peptide.	5

Supplementary Table 5 The yields of salivaricin B variants synthesized in UniBioCat reactions.

Variant	MW (Da)[a]	Yield (µM)	Variant	MW (Da)[a]	Yield (µM)
SalB_Q6A	2747	115	SalB_L20F-Q6A	2781	33
SalB_Q6C	2779	19	SalB_L20F-Q6H	2847	51
SalB_Q6E	2805	12	SalB_L20F-Q6K	2838	40
SalB_Q6F	2823	5	SalB_L20F-Q6M	2841	28
SalB_Q6G	2733	21	SalB_L20F-Q6N	2824	25
SalB_Q6H	2813	26	SalB_L20I-Q6A	2765	35
SalB_Q6I	2789	13	SalB_L20I-Q6H	2831	31
SalB_Q6K	2804	32	SalB_L20I-Q6K	2822	30
SalB_Q6L	2789	23	SalB_L20I-Q6M	2825	48
SalB_Q6M	2807	23	SalB_L20I-Q6N	2825	84
SalB_Q6N	2790	50	SalB_L20M-Q6A	2747	55
SalB_Q6S	2763	44	SalB_L20M-Q6H	2813	54
SalB_Q6V	2775	23	SalB_L20M-Q6K	2804	33
SalB_Q6W	2862	9	SalB_L20M-Q6M	2807	31
SalB_Q6Y	2839	25	SalB_L20M-Q6N	2790	59
SalB_L20A	2762	30			
SalB_L20C	2794	15			
SalB_L20E	2820	22			
SalB_L20F	2838	33			
SalB_L20H	2828	39			
SalB_L20I	2804	71			
SalB_L20K	2819	21			
SalB_L20M	2822	65			
SalB_L20N	2805	93			
SalB_L20Q	2819	12			
SalB_L20R	2847	16			
SalB_L20S	2778	15			
SalB_L20V	2790	44			
SalB_L20W	2877	52			
SalB_L20Y	2854	14			

[a] MW, molecular weight.

Supplementary Table 6 Minimal inhibitory concentrations (MIC) and IC_{50} of salivaricin B and variants.

**Peptides**	***Bacillus subtilis***		***Micrococcus luteus***		***Lactococcus lactis***	
	**MIC (μM)**	**IC_{50} (μM)**	**MIC (μM)**	**IC_{50} (μM)**	**MIC (μM)**	**IC_{50} (μM)**
Salivaricin B	4	1.88±0.01	2	1.19±0.05	4	1.67±0.08
SalB_Q6H	1	0.52±0.03	0.5	0.34±0.05	1	0.58±0.10
SalB_L20M	4	1.82±0.05	1	0.58±0.02	4	1.97±0.03
SalB_L20I-Q6H	2	1.56±0.30	2	0.84±0.17	2	1.07±0.13
SalB_L20M-Q6H	4	1.83±0.12	2	1.19±0.10	4	1.71±0.17

Source data are provided as a Source Data file.

Supplementary Figures

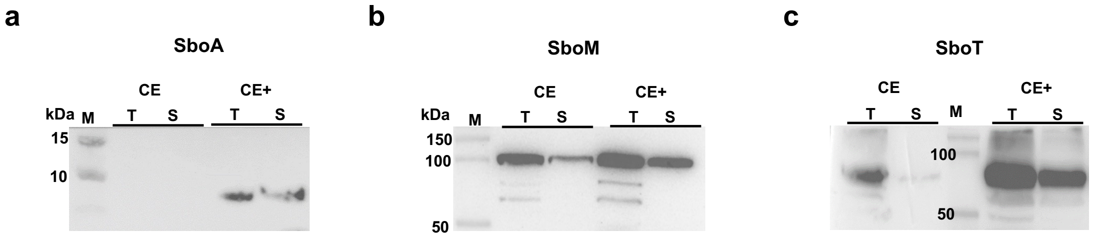

Supplementary Figure 1. Cell-free expression of salivaricin B biosynthesis genes.
Western-blot analysis of cell-free expressed (**a**) SboA (7.8 kDa), (**b**) SboM (113 kDa), and (**c**) SboT (83 kDa). Peptide/enzymes are labeled with the anti-His antibody. CE, cell extracts prepared from *E. coli* BL21 Star (DE3); CE+, CE enriched with chaperones (DanK-DnaJ-GrpE and GroES-GroEL); M, protein marker; T, total protein; S, soluble protein. Results were reproduced three times independently; representative data are shown. Source data are provided as a Source Data file.

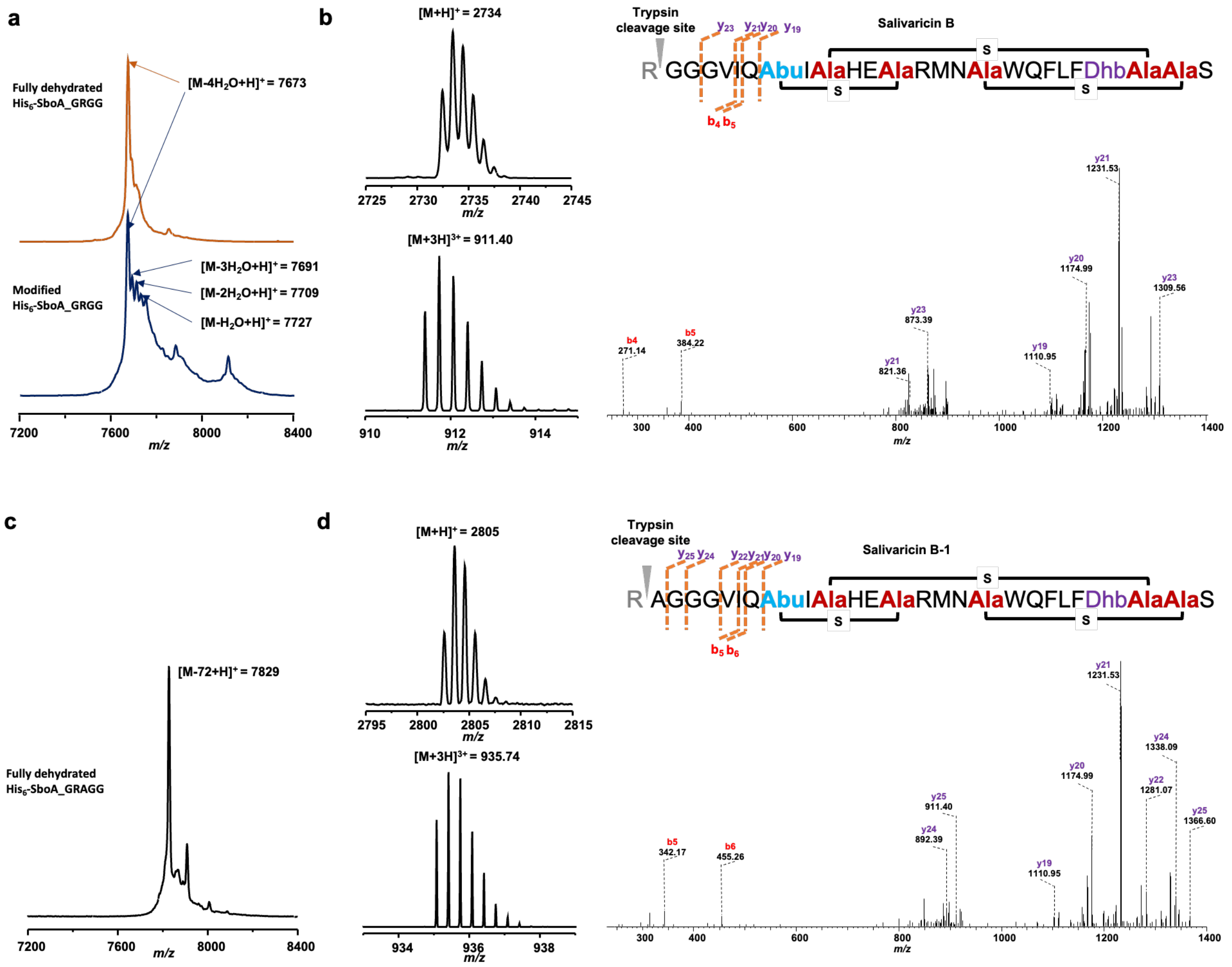

Supplementary Figure 2. ***In vivo*** **biosynthesis and identification of salivaricin B and salivaricin B-1.** (**a**) MALDI-TOF-MS analysis of modified precursor peptide of SboA_A-1R. (**b**) MALDI-TOF-MS (top left), LC-MS (bottom left), and MS/MS (right) analysis of matured salivaricin B (*in vitro* maturation by trypsin digestion). (**c**) MALDI-TOF-MS analysis of fully dehydrated precursor peptide of SboA_G-2R. (**d**) MALDI-TOF-MS (top left), LC-MS (bottom left), and MS/MS (right) analysis of matured salivaricin B-1 (*in vitro* maturation by trypsin digestion).

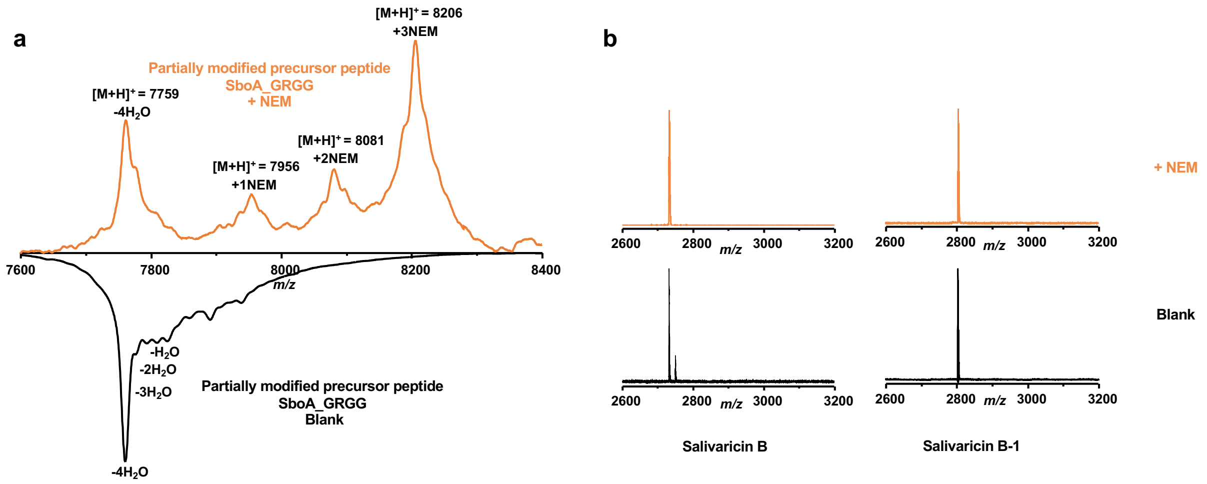

Supplementary Figure 3. Identification of the thioether formation by using the *N*-ethylmaleimide (NEM) derivatization method.

(**a**) MALDI-TOF-MS (linear positive mode) identification of partially modified precursor peptide SboA_A-1R treated with NEM. Note that the masses indicated as +1NEM, +2NEM, and +3NEM are non-dehydrated peptide peaks. (**b**) MALDI-TOF-MS (reflectron positive mode) identification of mature salivaricin B and salivaricin B-1. In the alkylation assay, NEM is added to the thiol (-SH) group of the cysteine residue and the derivatives show a molecular mass increase of 125 Da per reaction. The partially modified SboA_A-1R was a peptide mixture purified from the *in vivo* co-expression of sboA_A-1R and sboM for 1 h at 30°C, in which serine and threonine residues were not completely dehydrated to form the thioether crosslinks leaving 1-3 cysteine residues to react with NEM. When NEM was added, the peptide molecular mass increased accordingly. For the fully modified peptide, no NEM was added and thus the peptide molecular mass was not changed as observed in (**b**) for both salivaricin B and salivaricin B-1. The consistent molecular weights indicate the successful modification of the thioether crosslinks in the mature peptides.

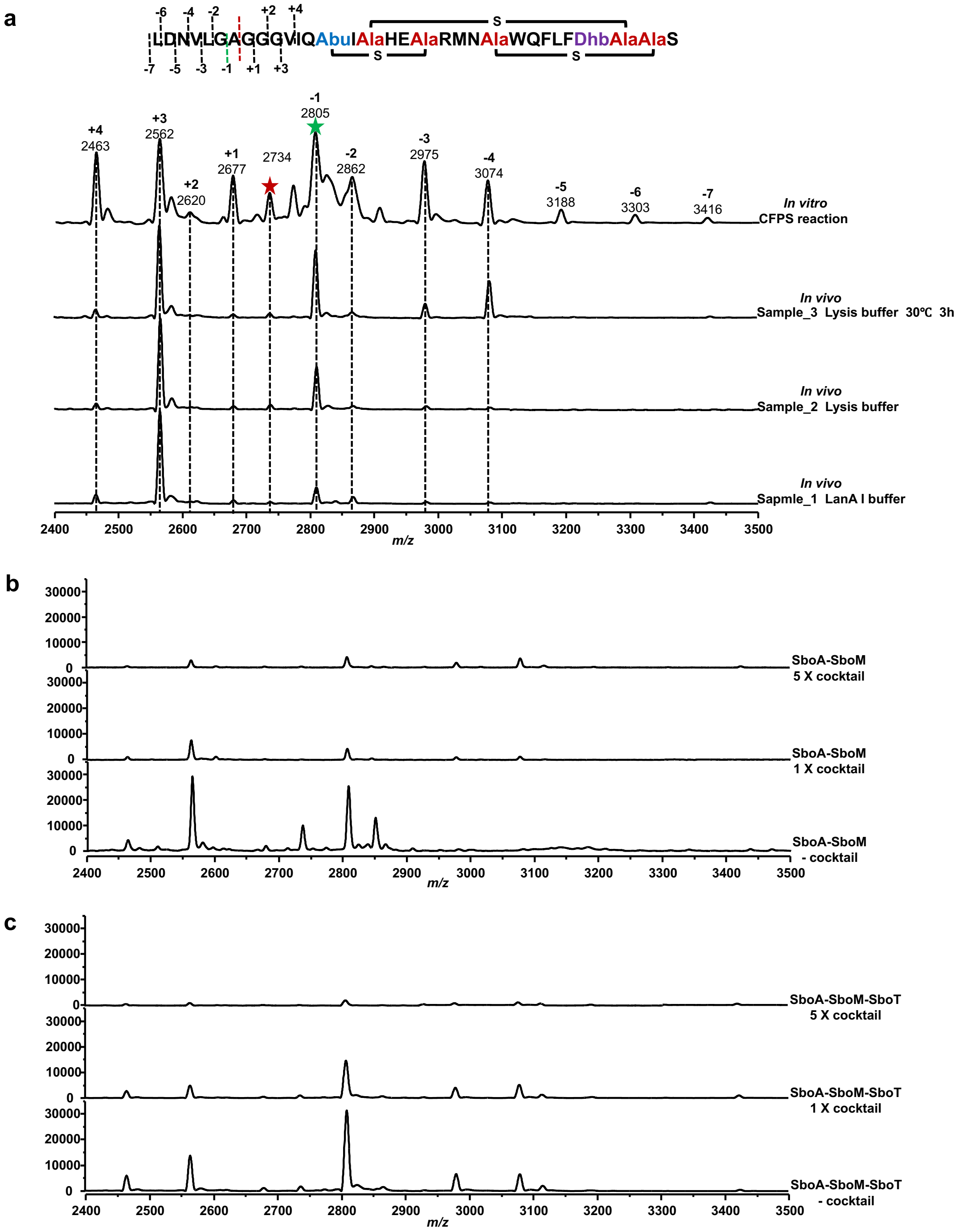

Supplementary Figure 4. Effects of endogenous potential proteases on salivaricin B synthesis.
(**a**) Generation of salivaricin B analogs by non-specific protease degradation both *in vivo* and *in vitro*. The red and green stars indicate salivaricin B and salivaricin B-1, respectively. Analogs detected *in vitro* were products from cell-free co-expression of sboA, sboM, and sboT. Analogs detected *in vivo* were products from co-expression of sboA and sboM. Harvested strains were divided into three parts undergoing different treatments. Sample 1 was suspended in LanA I buffer containing 4 M guanidine hydrochloride and then sonicated for cell lysis, in which the endogenous proteases were inactivated by guanidine hydrochloride. Sample 2 was suspended

in lysis buffer, sonicated, and then immediately placed on ice. Sample 3 was suspended in lysis buffer and incubated at 30°C for 3 h after sonication, in which the endogenous proteases conducted catalysis. All of samples were then centrifuged and the supernatants were desalted by C18 ZipTip and analyszed by MALDI-TOF-MS with linear positive mode. (**b**) Products from cell-free reactions (SboA and SboM) with increased concentrations of protein inhibitor cocktail in the UniBioCat reactions. (**c**) Products from cell-free reactions (SboA, SboM, and SboT) with increased concentrations of protein inhibitor cocktail in the UniBioCat reactions. The catalytic activities of endogenous proteases/peptidases and SboT were inhibited by a high concentration of protein inhibitor (5x).

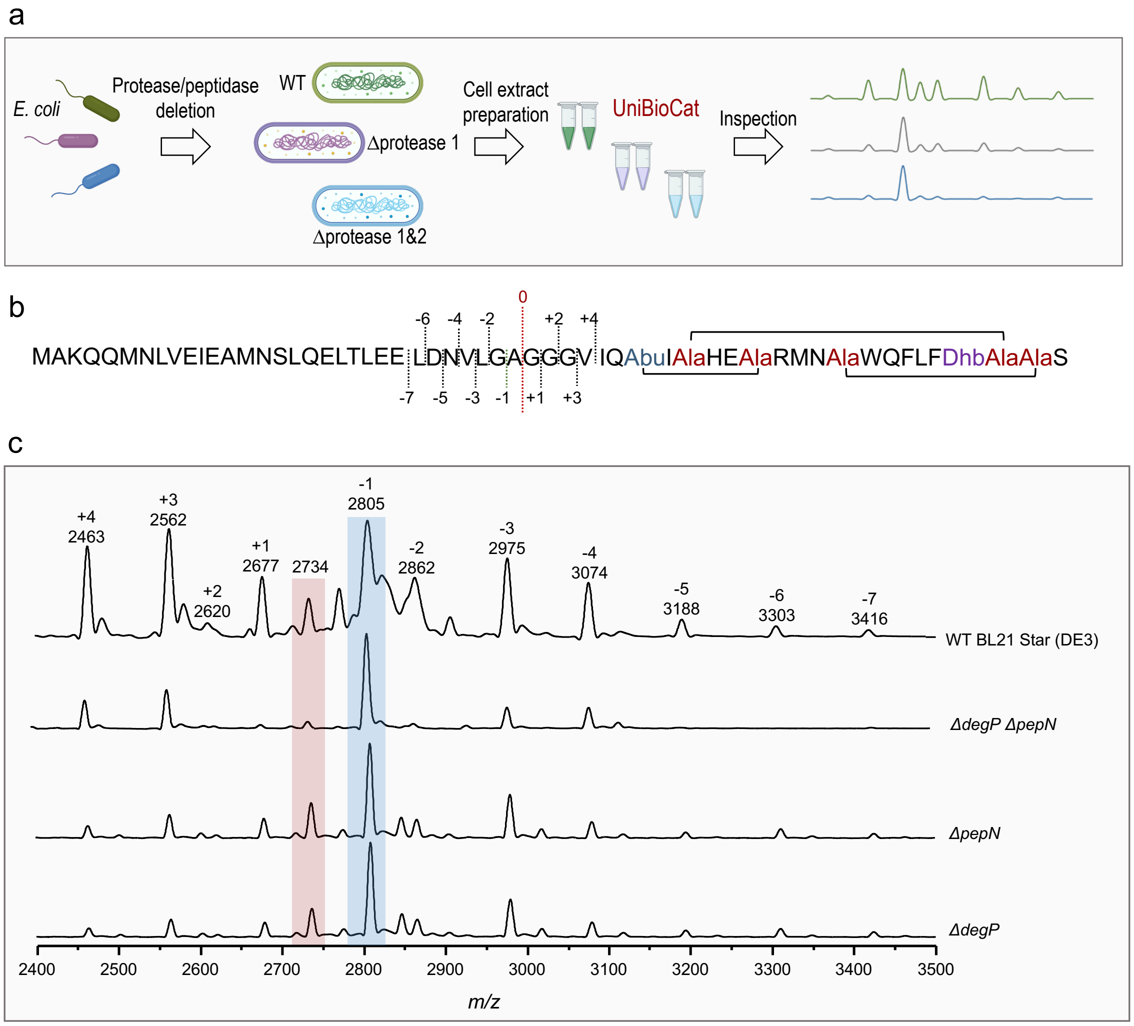

Supplementary Figure 5 Strain engineering to reduce non-specific protease/peptidase degradation in UniBioCat reactions.

(**a**) Schematic diagram of protease/peptidase deletion for the preparation of enhanced cell extracts for UniBioCat reactions. Created with BioRender.com. (**b**) Observed products by non-specific degradation with endogenous proteases and/or peptidases. (**c**) MALDI-TOF-MS analysis of salivaricin B (highlighted in red), salivaricin B-1 (highlighted in blue), and other non-specifically degraded products. Cell extracts used for UniBioCat were prepared from the wild-type (WT) strain *E. coli* BL21 Star (DE3) and three other engineered strains with protease (*degP*) and/or peptidase (*pepN*) gene(s) deleted. Note that SboM was pre-expressed in each engineered strain for the preparation of SboM-enriched cell extracts, which were then used for cell-free co-expression of SboA and SboT.

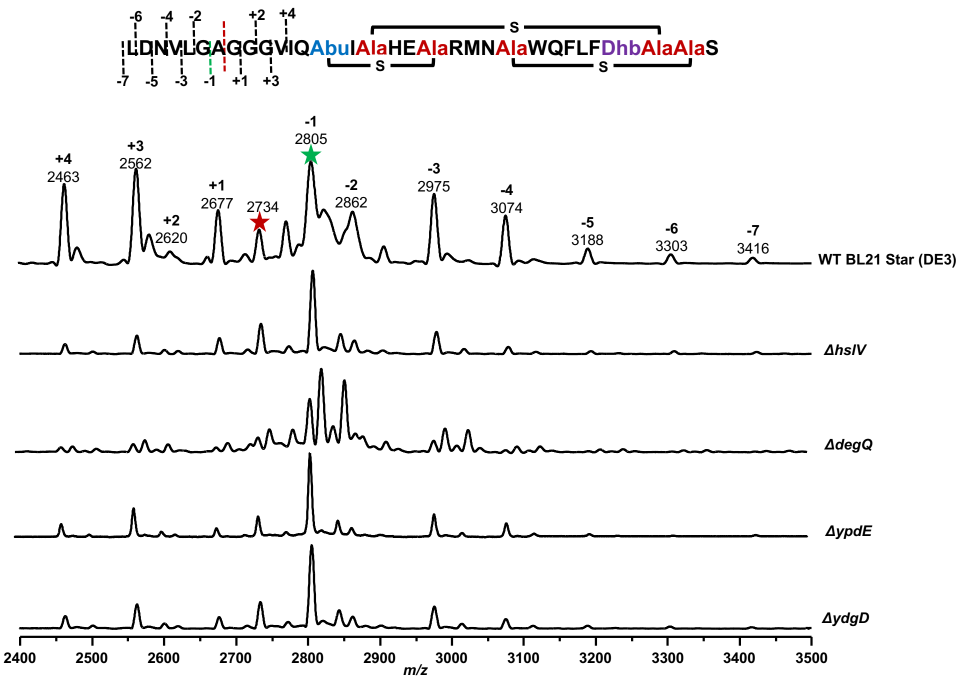

Supplementary Figure 6. Reduction of the non-specific protease/peptidase degradation in UniBioCat reactions.

Top, observed products by non-specific degradation with endogenous proteases/peptidases. Bottom, MALDI-TOF-MS analysis of salivaricin B (red star), salivaricin B-1 (blue star), and other non-specifically degraded products. Cell extracts used for UniBioCat were prepared from the wild-type (WT) strain *E. coli* BL21 Star (DE3) and four engineered strains with protease or peptidase deleted.

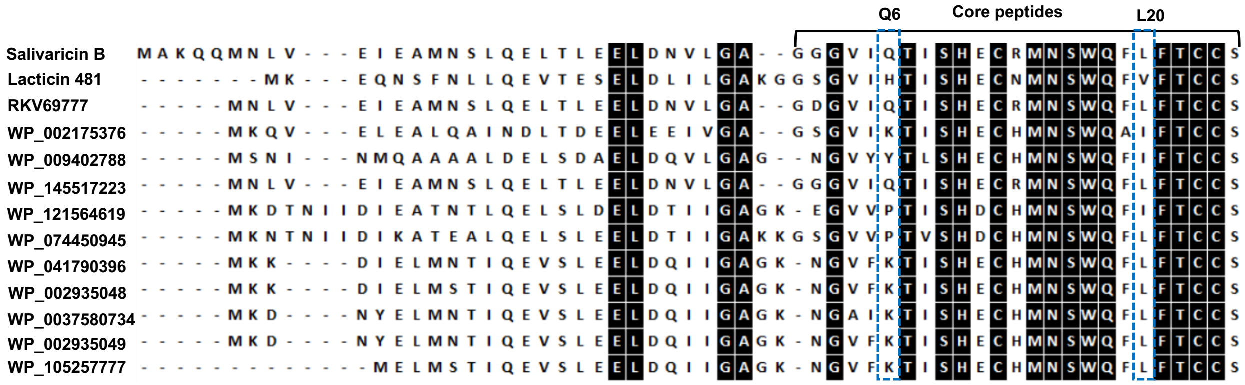

Supplementary Figure 7. Alignment of homologous peptides based on the salivaricin B precursor peptide. Amino acid sequences of the homologous peptides were aligned by using MEGA. Q6 and L20 were selected for the mutation.

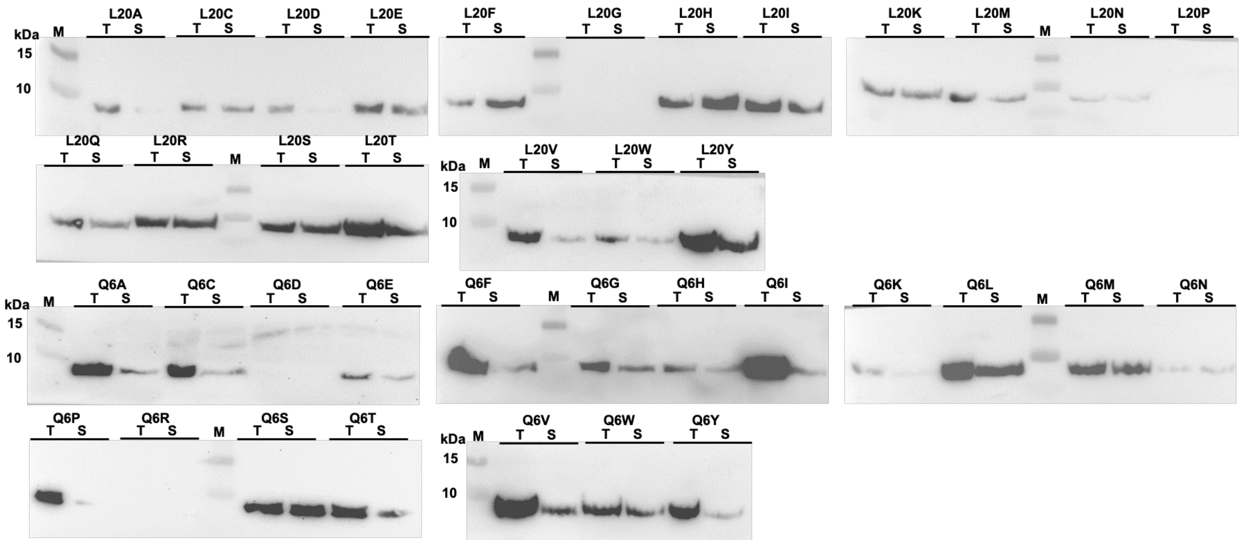

Supplementary Figure 8. Western-blot analysis of cell-free expressed mutated precursor peptides. Soluble peptides: SalB_L20C, SalB_L20E, SalB_L20F, SalB_L20H, SalB_L20I, SalB_L20K, SalB_L20M, SalB_L20N, SalB_L20Q, SalB_L20R, SalB_L20S, SalB_L20T, SalB_L20V, SalB_L20W, SalB_L20Y, SalB_Q6A, SalB_Q6C, SalB_Q6E, SalB_Q6F, SalB_Q6G, SalB_Q6H, SalB_Q6L, SalB_Q6M, SalB_Q6N, SalB_Q6S, SalB_Q6T, SalB_Q6V, SalB_Q6W, and SalB_Q6Y. Insoluble peptides: SalB_L20A, SalB_L20D, SalB_Q6I, SalB_Q6K, and SalB_Q6P. Non-expressed peptides: SalB_L20G, SalB_L20P, SalB_Q6D, and SalB_Q6R. Peptides are labeled with the anti-His antibody. Results were reproduced three times independently; representative data are shown. Source data are provided as a Source Data file.

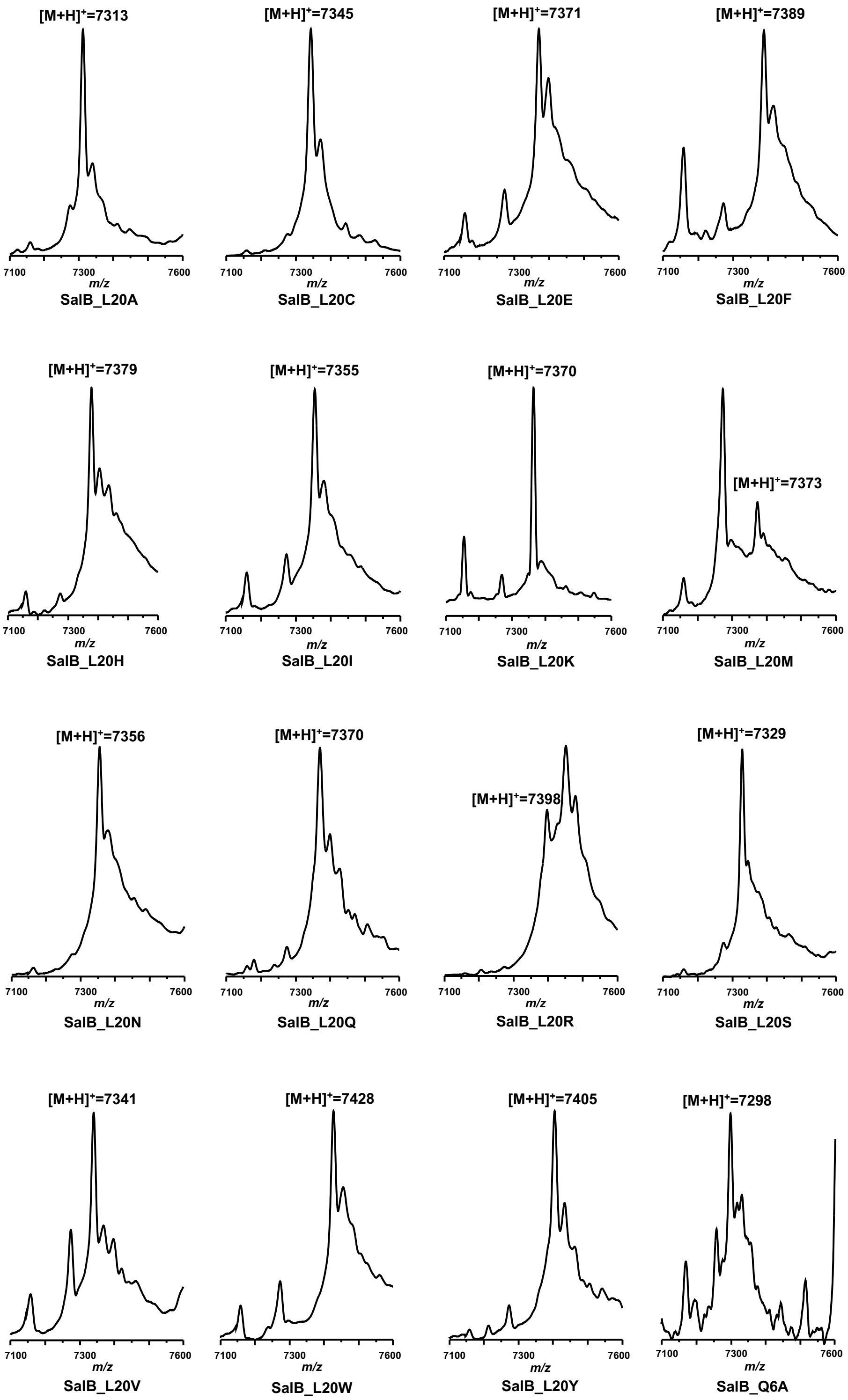
[M+H]+=7313
SalB_L20A
[M+H]+=7345
SalB_L20C
[M+H]+=7371
SalB_L20E
[M+H]+=7389
SalB_L20F
[M+H]+=7379
SalB_L20H
[M+H]+=7355
SalB_L20I
[M+H]+=7370
SalB_L20K
[M+H]+=7373
SalB_L20M
[M+H]+=7356
SalB_L20N
[M+H]+=7370
SalB_L20Q
[M+H]+=7398
SalB_L20R
[M+H]+=7329
SalB_L20S
[M+H]+=7341
SalB_L20V
[M+H]+=7428
SalB_L20W
[M+H]+=7405
SalB_L20Y
[M+H]+=7298
SalB_Q6A
7100
7300
7600
m/z

(*continued*)

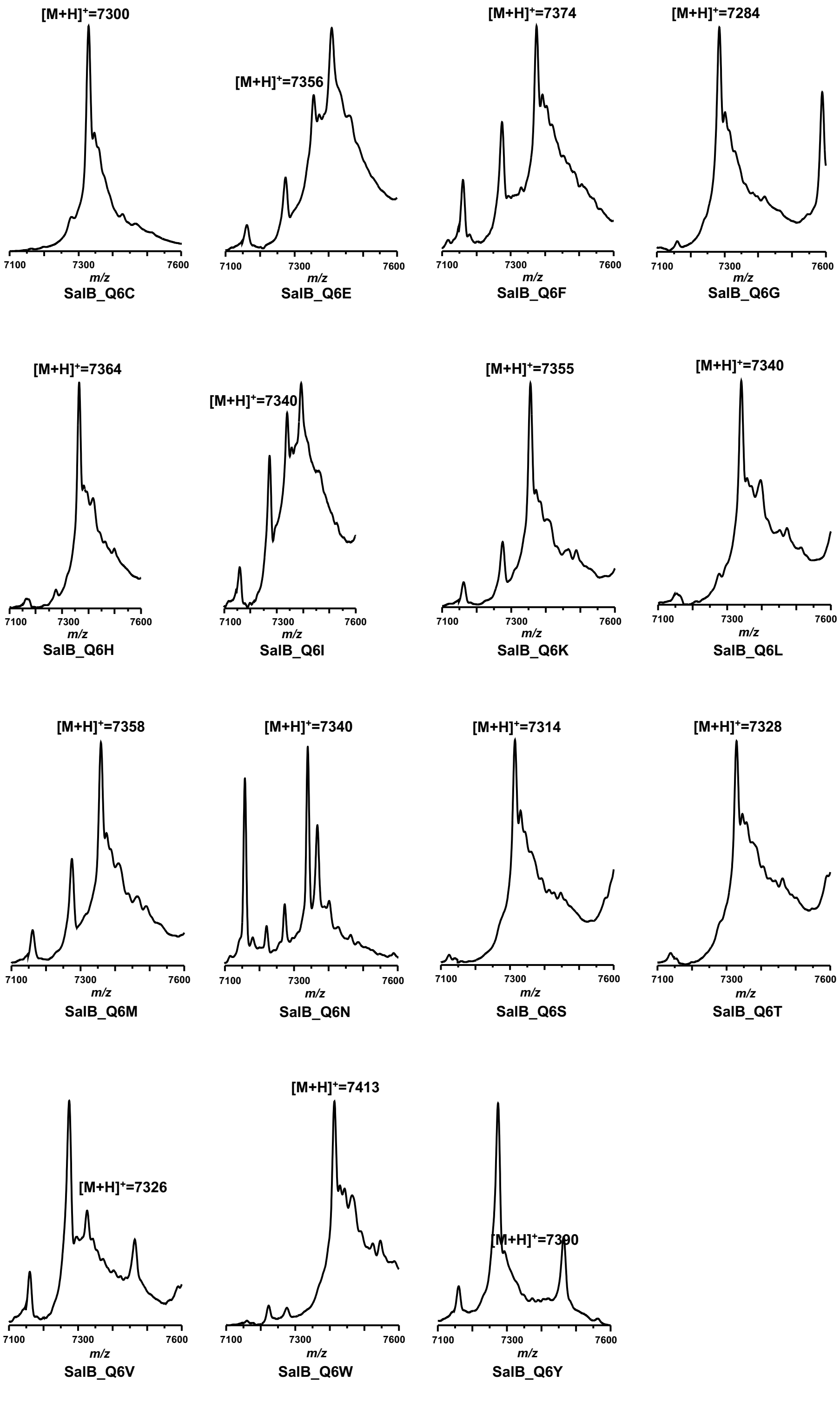

Supplementary Figure 9. MALDI-TOF-MS analysis of cell-free expressed mutated precursor peptides. The products were purified and identified by MALDI-TOF-MS with linear positive mode. The mass labels correspond to the $[M+H]^+$ ion of the synthesized products.

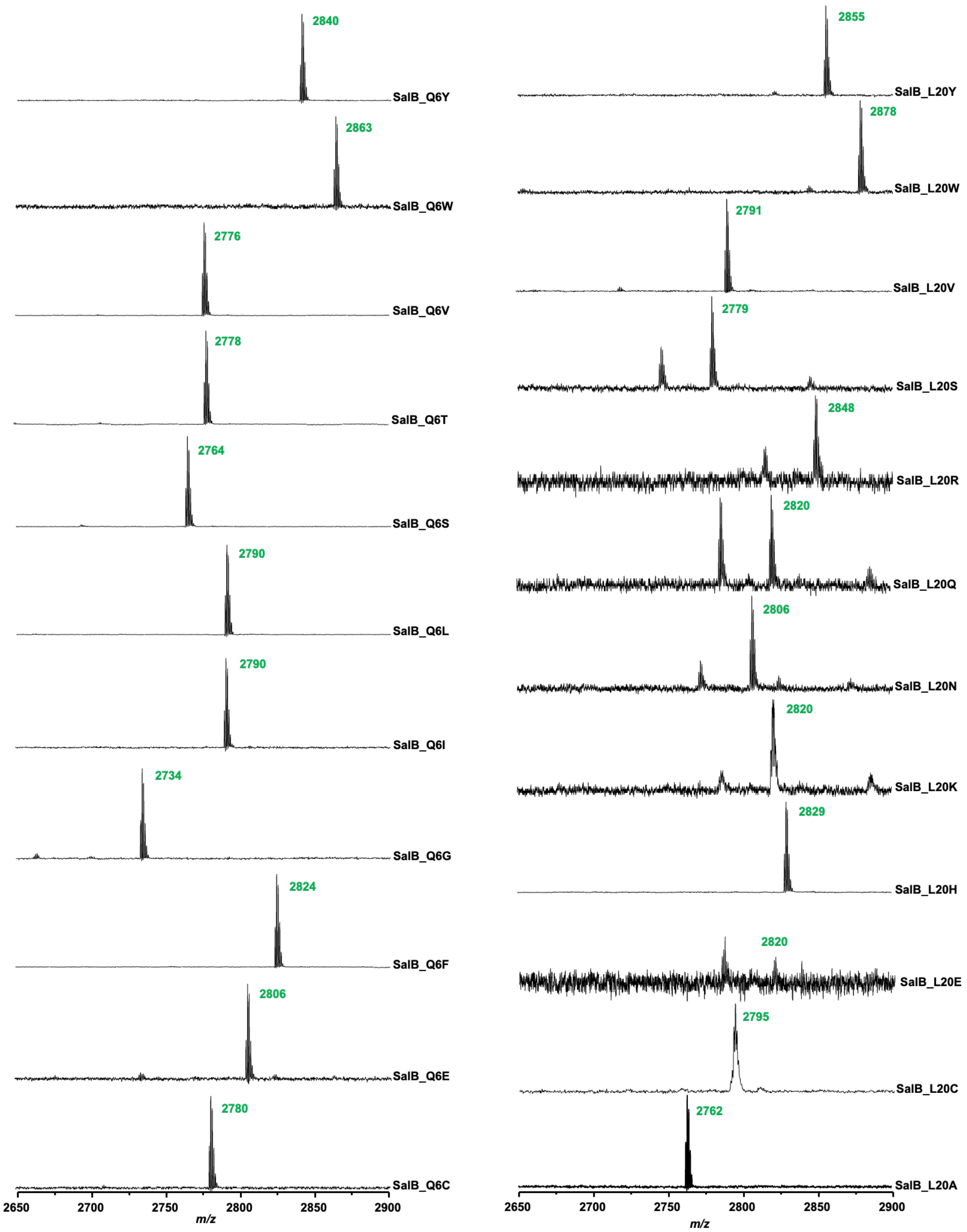

Supplementary Figure 10. MALDI-TOF-MS analysis of salivaricin B mutants (mutated at Q6 or L20). Products were identified by MALDI-TOF-MS with reflectron positive mode. The mass labels correspond to the $[M+H]^+$ ion of the mutant peptides derived from salivaricin B-1 (note that salivaricin B-1 based variants are the most abundant products in all analyses).

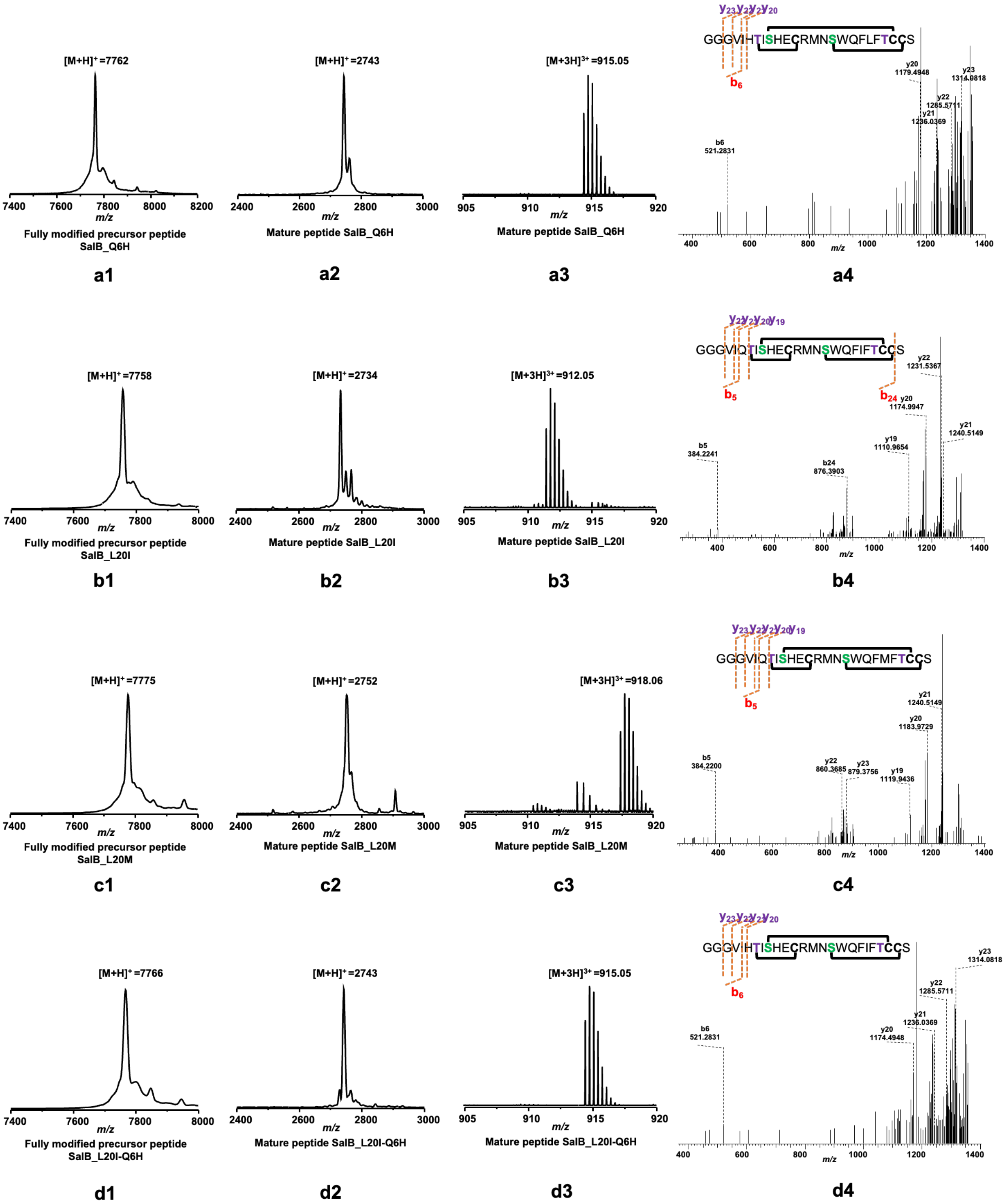

[M+H]+ =7762
[M+H]+ =2743
[M+3H]3+ =915.05
GGGVIHTISHECRMNSWQFLFTCCS
Fully modified precursor peptide SalB_Q6H
Mature peptide SalB_Q6H
Mature peptide SalB_Q6H
a1
a2
a3
a4
[M+H]+ =7758
[M+H]+ =2734
[M+3H]3+ =912.05
GGGVIQTISHECRMNSWQFIFTCCS
Fully modified precursor peptide SalB_L20I
Mature peptide SalB_L20I
Mature peptide SalB_L20I
b1
b2
b3
b4
[M+H]+ =7775
[M+H]+ =2752
[M+3H]3+ =918.06
GGGVIQTISHECRMNSWQFMFTCCS
Fully modified precursor peptide SalB_L20M
Mature peptide SalB_L20M
Mature peptide SalB_L20M
c1
c2
c3
c4
[M+H]+ =7766
[M+H]+ =2743
[M+3H]3+ =915.05
GGGVIHTISHECRMNSWQFIFTCCS
Fully modified precursor peptide SalB_L20I-Q6H
Mature peptide SalB_L20I-Q6H
Mature peptide SalB_L20I-Q6H
d1
d2
d3
d4

(*continued*)

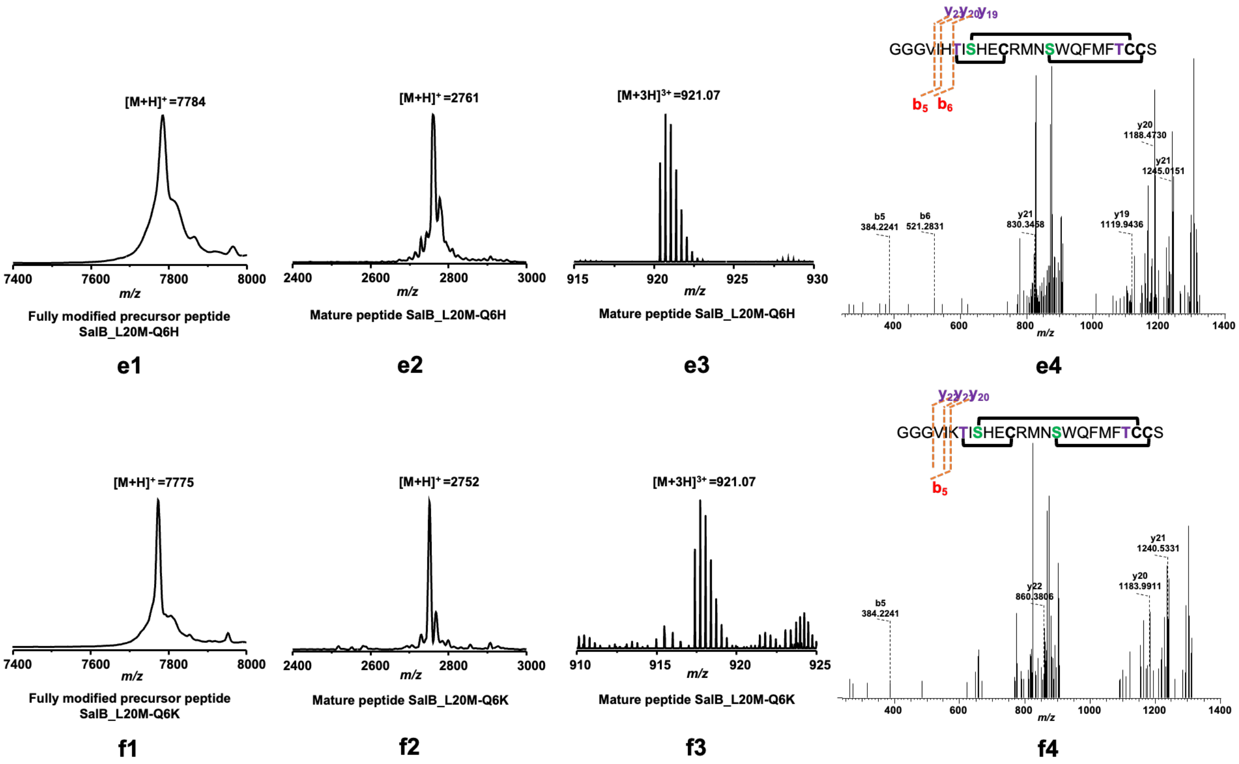

Supplementary Figure 11. Identification of *in vivo* synthesized salivaricin B variants.

(**a1-f1**) Fully modified precursor peptides identified by MALDI-TOF-MS with linear positive mode. (**a2-f2**) Mature peptides identified by MALDI-TOF-MS with linear positive mode. (**a3-f3**) Mature peptides identified by LC-MS. (**a4-f4**) MS/MS analysis of mature peptides.

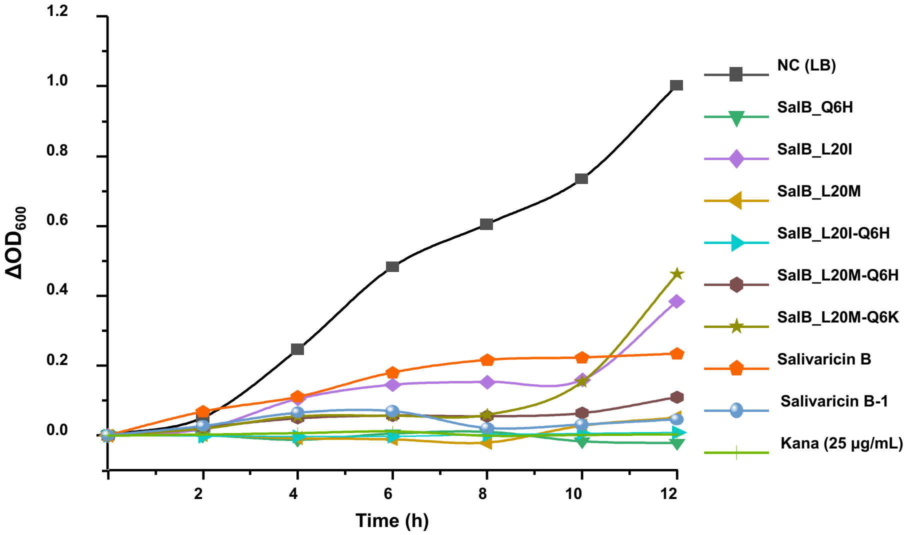

Supplementary Figure 12. Antimicrobial activity assay of selected salivaricin B variants. Antimicrobial activity assay showing ΔOD_{600} profiles of *S. aureus* RN4220 growth treated with selected single- and two-site mutants. These tested mutants were synthesized and modified *in vivo*, followed by purification and *in vitro* maturation with trypsin digestion. In each activity assay, 50 µM of each pure mutant was added to the cultivation. NC, negative control by adding an equal volume of LB medium to the cultivation. Kanamycin (Kana) is used as a positive control. The data were obtained from one experiment (n = 1). Source data are provided as a Source Data file.

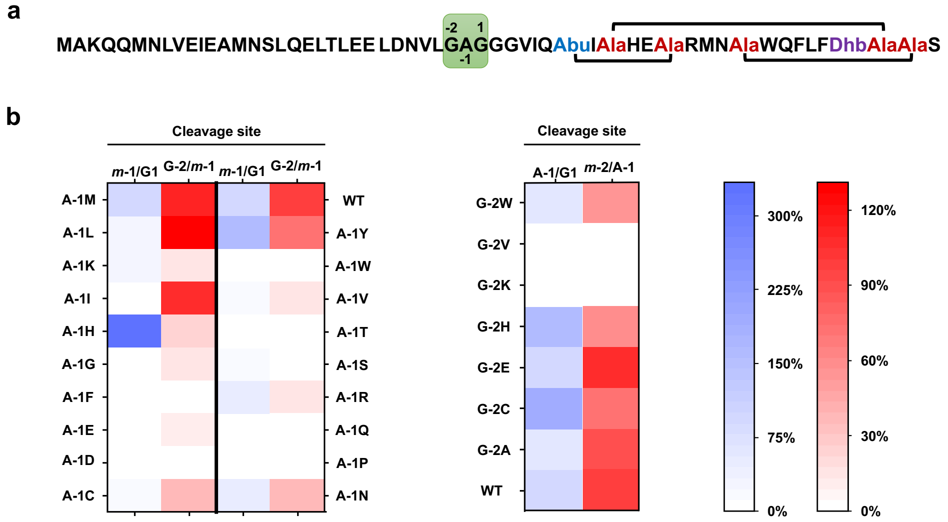

Supplementary Figure 13. Evaluating substrate tolerance of SboT (cleavage site "GAG" is highlighted). (**a**) SboM modified precursors (SboA) serving as substrates for SboT. The position of A-1 was selected for saturation mutagenesis. The position of G-2 was mutated to seven other amino acids (i.e., W, V, K, H, E, C, and A). (**b**) Effect of specific-site mutation (A-1 or G-2) on the cleavage site of SboT. Heatmap analysis indicates the relative intensity (%) of mutated products to wild-type peptides (the percent values are calculated according to the mass peaks observed by MALDI-TOF-MS, see **Supplementary Figure 14**). *m*, mutated amino acids at the position of -1 or -2; WT, wild-type SboA without mutation. Source data are provided as a Source Data file.

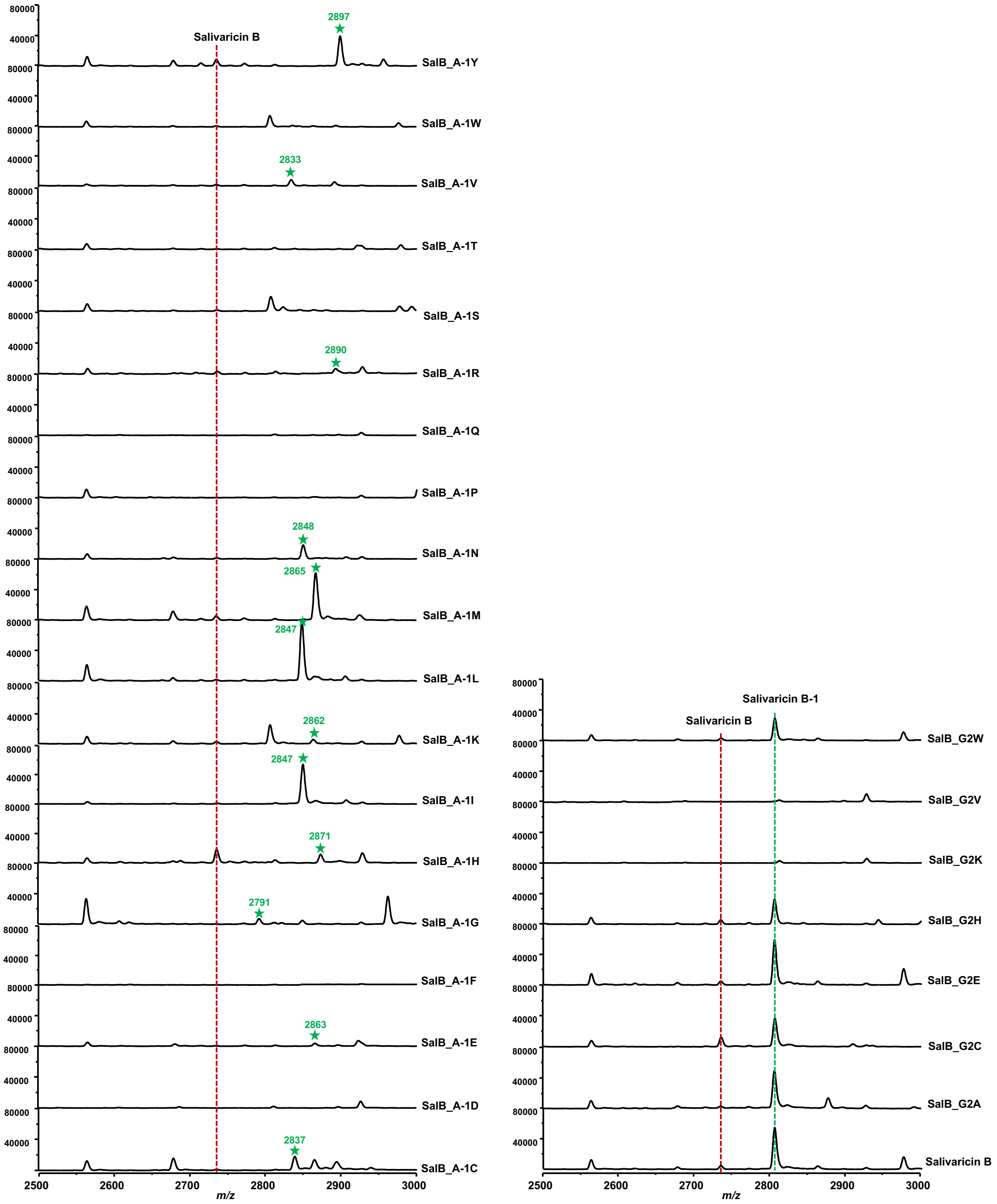

Supplementary Figure 14. MALDI-TOF-MS analysis of peptides matured by SboT.

Products were identified by MALDI-TOF-MS with linear positive mode. Red lines indicate salivaricin B resulted from cleavage site of mutant-1/G and A-1/G. Green stars indicate products resulted from cleavage site of G-2/mutant. Green line indicates salivaricin B-1 resulted from cleavage site of mutant-2/A-1. See **Supplementary Figure 13** for the cleavage site “GAG” of SboT and mutated positions.

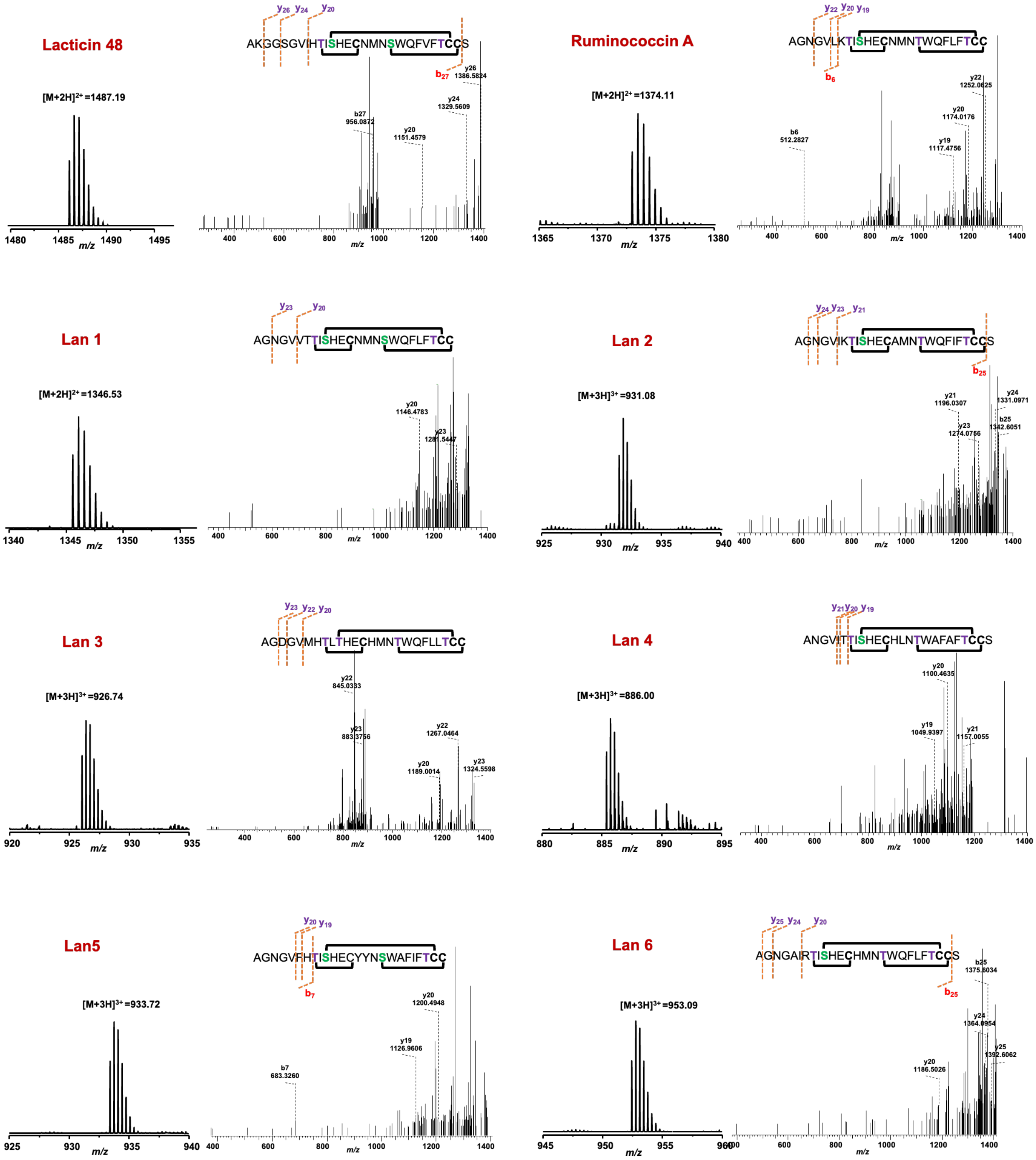
Lacticin 48
AKGGSGVIHTISHECNMNSWQFVFTCCS
[M+2H]2+ =1487.19
b27 956.0872
y20 1151.4579
y24 1329.5609
y26 1386.5824
Ruminococcin A
AGNGVLKTISHECNMNTWQFLFTCC
[M+2H]2+ =1374.11
b6 512.2827
y19 1117.4756
y20 1174.0176
y22 1252.0625
Lan 1
AGNGVVTTISHECNMNSWQFLFTCC
[M+2H]2+ =1346.53
y20 1146.4783
Lan 2
AGNGVIKTISHECAMNTWQFIFTCCS
[M+3H]3+ =931.08
y21 1196.0307
y23 1274.0756
y24 1331.0971
b25 1342.6051
Lan 3
AGDGVMHTLTHECHMNTWQFLLTCC
[M+3H]3+ =926.74
y22 845.0333
y23 883.3756
y20 1189.0014
y22 1267.0464
y23 1324.5598
Lan 4
ANGVITTISHECHLNTWAFAFTCCS
[M+3H]3+ =886.00
y20 1100.4635
y19 1049.9397
y21 1157.0055
Lan5
AGNGVFHTISHECYYNSWAFIFTCC
[M+3H]3+ =933.72
b7 683.3260
y19 1126.9606
y20 1200.4948
Lan 6
AGNGAIRTISHECHMNTWQFLFTCCS
[M+3H]3+ =953.09
b25 1375.6034
y24 1364.0954
y25 1392.6062
y20 1186.5026
m/z

(*continued*)

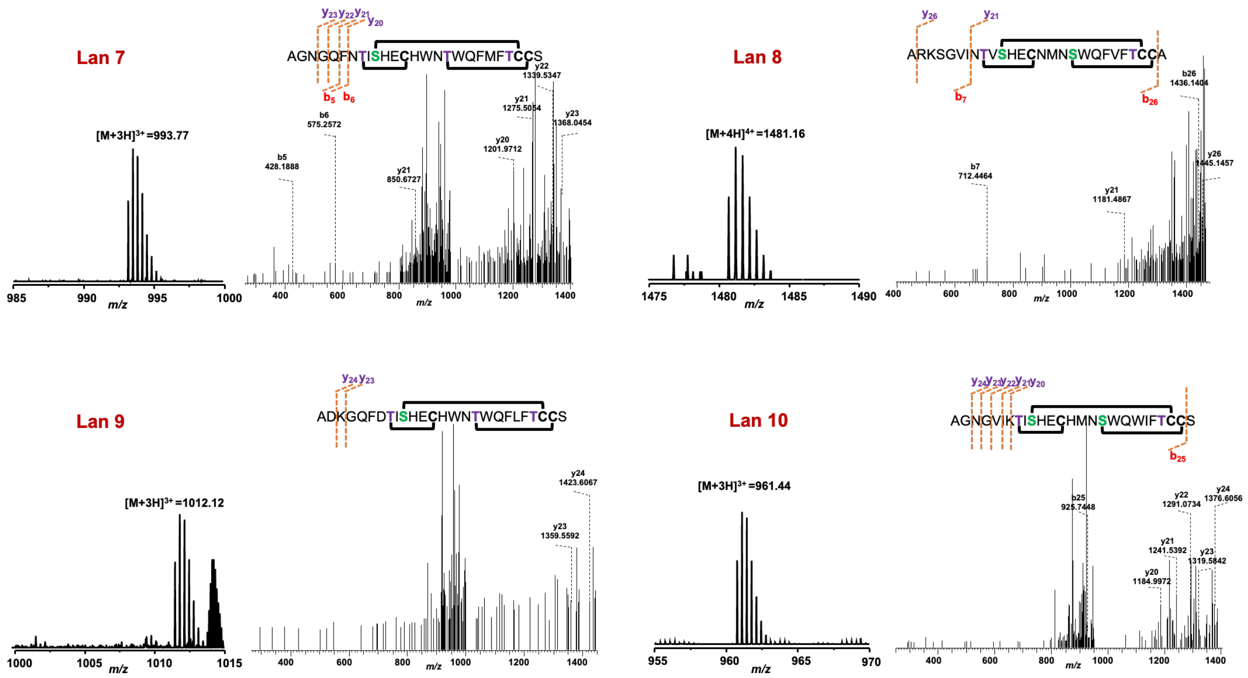

Supplementary Figure 15. LC-MS/MS analysis of two known (lacticin 481 and ruminococcin A) and ten uncharacterized lanthipeptides (Lan 1 – Lan 10).

All lanthipeptides were cell-free synthesized in UniBioCat reactions. Note that all sequences are presented with one additional amino acid alanine at the *N*-terminus of the final peptide.

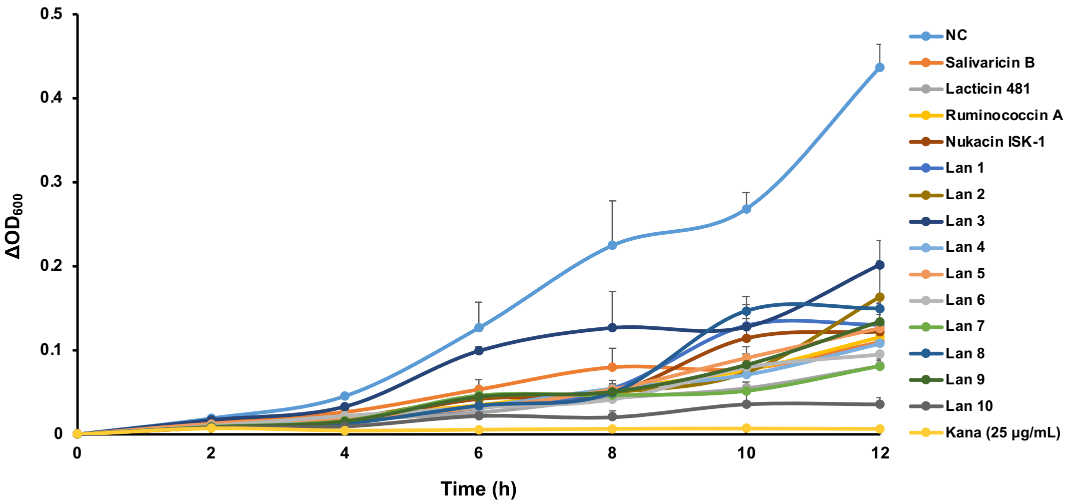

Supplementary Figure 16. Antimicrobial activity assay of ten uncharacterized lanthipeptides (Lan 1 – Lan 10).

Antimicrobial activity assay showing ΔOD_{600} profiles of *S. aureus* RN4220 growth treated with cell-free synthesized novel lanthipeptides. Note that cell-free reaction mixture is used directly for the assay without purification of each lanthipeptide. NC, negative control without gene templates in the cell-free reaction. Kanamycin (Kana) is used as a positive control. Data are presented as mean±s.d. of two independent experiments (n = 2). Source data are provided as a Source Data file.

Supplementary References

1. Kolmar, H., Waller, R. P. & Sauer, R. T. The DegP and DegQ periplasmic endoproteases of *Escherichia coli*: Specificity for cleavage sites and substrate conformation. *J. Bacteriol.* **178**, 5925-5929 (1996).
2. Page, M. J. & Di Cera, E. Serine peptidases: Classification, structure and function. *Cell. Mol. Life Sci.* **65**, 1220-1236 (2008).
3. Rohrwild, M., Coux, O., Huang, H. C., Moerschell, R. P., Yoo, S. J., Seol, J. H., Chung, C. H. & Goldberg, A. L. HslV-HslU: A novel ATP-dependent protease complex in *Escherichia coli* related to the eukaryotic proteasome. *Proc. Natl. Acad. Sci. U S A.* **93**, 5808-5813 (1996).
4. Chen, S., Xu, B., Chen, E., Wang, J., Lu, J., Donadio, S., Ge, H. & Wang, H. Zn-dependent bifunctional proteases are responsible for leader peptide processing of class III lanthipeptides. *Proc. Natl. Acad. Sci. U S A.* **116**, 2533-2538 (2019).
5. Zheng, Y., Roberts, R. J., Kasif, S. & Guan, C. Characterization of two new aminopeptidases in *Escherichia coli*. *J. Bacteriol.* **187**, 3671-3677 (2005).
