# Supplementary material for: Cell-free biosynthesis and engineering of ribosomally synthesized lanthipeptides
Source: Nat Commun. 2024 May 21;15:4336. doi: 10.1038/s41467-024-48726-y (PMC11109155; doi:10.1038/s41467-024-48726-y)
Supplement: Supplementary file 3 — Reporting Summary [file 41467_2024_48726_MOESM3_ESM.pdf]

nature portfolio

Corresponding author(s): Yifan Liu, Shengjie Ling, Michael C. Jewett, Jian Li

Last updated by author(s): Apr 22, 2024

Reporting Summary

Nature Portfolio wishes to improve the reproducibility of the work that we publish. This form provides structure for consistency and transparency in reporting. For further information on Nature Portfolio policies, see our Editorial Policies and the Editorial Policy Checklist.

Statistics

For all statistical analyses, confirm that the following items are present in the figure legend, table legend, main text, or Methods section.

n/a	Confirmed	
☐	☒	The exact sample size (*n*) for each experimental group/condition, given as a discrete number and unit of measurement
☐	☒	A statement on whether measurements were taken from distinct samples or whether the same sample was measured repeatedly
☒	☐	The statistical test(s) used AND whether they are one- or two-sided<br>*Only common tests should be described solely by name; describe more complex techniques in the Methods section.*
☒	☐	A description of all covariates tested
☒	☐	A description of any assumptions or corrections, such as tests of normality and adjustment for multiple comparisons
☐	☒	A full description of the statistical parameters including central tendency (e.g. means) or other basic estimates (e.g. regression coefficient) AND variation (e.g. standard deviation) or associated estimates of uncertainty (e.g. confidence intervals)
☒	☐	For null hypothesis testing, the test statistic (e.g. *F*, *t*, *r*) with confidence intervals, effect sizes, degrees of freedom and *P* value noted<br>*Give P values as exact values whenever suitable.*
☒	☐	For Bayesian analysis, information on the choice of priors and Markov chain Monte Carlo settings
☒	☐	For hierarchical and complex designs, identification of the appropriate level for tests and full reporting of outcomes
☒	☐	Estimates of effect sizes (e.g. Cohen's *d*, Pearson's *r*), indicating how they were calculated

Our web collection on statistics for biologists contains articles on many of the points above.

Software and code

Policy information about availability of computer code

Data collection	Microplate reader (BioTek Gen5) was used for OD600 measurements; UVP ChemStudio (analytikjena) was used for imaging Western-blot analysis; matrix-assisted laser desorption/ionization time-of-flight mass spectrometry (MALDI-TOF-MS; Bruker, Autoflex Speed) and liquid chromatography-tandem mass spectrometry (LC-MS/MS; ThermoFisher, High Resolution) were used for peptide analysis; high performance liquid chromatography (HPLC, Agilent 1260 Infinity II) was used for peptide purification.
Data analysis	DNA sequences were analyzed by SnapGene viewer (version 4.3.10); MALDI-TOF-MS data were analyzed by FlexAnalysis software (version 3.4); LC-MS/MS data were analyzed by Xcalibur software (version 3.0.63); phylogenesis was analyzed by MEGA (version 7.0); IC50 values were determined by GraphPad Prism (version 10.2.1); Microsoft Excel and OriginPro (version 9.7.0.185) were used for general data analysis.

For manuscripts utilizing custom algorithms or software that are central to the research but not yet described in published literature, software must be made available to editors and reviewers. We strongly encourage code deposition in a community repository (e.g. GitHub). See the Nature Portfolio guidelines for submitting code & software for further information.

Data

Policy information about availability of data

All manuscripts must include a data availability statement. This statement should provide the following information, where applicable:

- Accession codes, unique identifiers, or web links for publicly available datasets
- A description of any restrictions on data availability
- For clinical datasets or third party data, please ensure that the statement adheres to our policy

All data supporting the findings of this study are available within the article and its Supplementary Information, or available from the corresponding author upon request. The source data underlying Fig. 2b and 2f, Fig. 3d, Fig. 4b, Supplementary Figs. 1, 8, 12, 13, and 16, and Supplementary Table 6 are provided as a Source Data file. Source data are provided with this paper.

Research involving human participants, their data, or biological material

Policy information about studies with human participants or human data. See also policy information about sex, gender (identity/presentation), and sexual orientation and race, ethnicity and racism.

Reporting on sex and gender	N/A
Reporting on race, ethnicity, or other socially relevant groupings	N/A
Population characteristics	N/A
Recruitment	N/A
Ethics oversight	N/A

Note that full information on the approval of the study protocol must also be provided in the manuscript.

Field-specific reporting

Please select the one below that is the best fit for your research. If you are not sure, read the appropriate sections before making your selection.

[x] Life sciences [ ] Behavioural & social sciences [ ] Ecological, evolutionary & environmental sciences

For a reference copy of the document with all sections, see nature.com/documents/nr-reporting-summary-flat.pdf

Life sciences study design

All studies must disclose on these points even when the disclosure is negative.

Sample size	All sample sizes used are listed in the manuscript. Western-blot analysis (Figure 2b) was performed three times independently. To analyze peptides using MALDI-TOF-MS and LC-MS, at least three independent experiments were performed. Antimicrobial activity assay (Figures 3d and 4b) in 96-well plates was carried out as n=2 as highly reproducible in repeated experiments and small differences in levels would not impact the conclusions.
Data exclusions	No data were excluded.
Replication	All experiments were performed as biological triplicates, unless stated otherwise. All attempts at replication were successful.
Randomization	Samples were organized by experimental variables then characterized fully and reported in completion. Therefore, samples were not randomized.
Blinding	We were not blinded to our experiments and analysis, because investigators needed to know which samples were included in the mass analysis and antimicrobial activity assay. Thus, blinding was not relevant to our study.

Reporting for specific materials, systems and methods

We require information from authors about some types of materials, experimental systems and methods used in many studies. Here, indicate whether each material, system or method listed is relevant to your study. If you are not sure if a list item applies to your research, read the appropriate section before selecting a response.

Materials & experimental systems

n/a	Involved in the study	
☐	☒	Antibodies
☒	☐	Eukaryotic cell lines
☒	☐	Palaeontology and archaeology
☒	☐	Animals and other organisms
☒	☐	Clinical data
☒	☐	Dual use research of concern
☒	☐	Plants

Methods

n/a	Involved in the study	
☒	☐	ChIP-seq
☒	☐	Flow cytometry
☒	☐	MRI-based neuroimaging

Antibodies

Antibodies used

Antibodies were used to visualize His-tag labeled peptides and enzymes in Western-blot analysis. The primary antibody was His-Tag Mouse Monoclonal Antibody (catalog number: 66005-1-Ig, 1:10000 dilution, Proteintech). The secondary antibody was HRP-conjugated Affinipure Goat Anti-Mouse IgG(H+L) (catalog number: SA00001-1, 1:10000 dilution, Proteintech).

Validation

The primary antibody His-Tag Mouse Monoclonal Antibody (catalog number: 66005-1-Ig, 1:10000 dilution, Proteintech) is originated from the host mouse. This antibody can be used in different applications including Western-blot analysis.

Plants

Seed stocks

N/A

Novel plant genotypes

N/A

Authentication

N/A
